# Amniogenic somatopleure: a novel origin of multiple cell lineages contributing to the cardiovascular system

**DOI:** 10.1038/s41598-017-08305-2

**Published:** 2017-08-21

**Authors:** Rieko Asai, Yuka Haneda, Daiki Seya, Yuichiro Arima, Kimiko Fukuda, Yukiko Kurihara, Sachiko Miyagawa-Tomita, Hiroki Kurihara

**Affiliations:** 1 Department of Physiological Chemistry and Metabolism, Graduate School of Medicine, The University of Tokyo, 7-3-1 Hongo, Bunkyo-ku, Tokyo 113-0033 Japan; 2 Core Research for Evolutional Science and Technology (CREST), Japan Science and Technology Agency (JST), Chiyoda-ku, Tokyo 102-0076 Japan; 3 Department of Pediatric Cardiology, Tokyo Women’s Medical University, 8-1 Kawada-cho, Shinjuku-ku, Tokyo 162-8666 Japan; 4 Department of Biological Sciences, Graduate School of Science, The University of Tokyo, 7-3-1 Hongo, Bunkyo-ku, Tokyo 113-0033 Japan; 5 Department of Biological Science, Tokyo Metropolitan University, 1-1 Minami-osawa, Hachioji, Tokyo 192-0397 Japan; 6 Department of Veterinary Technology, Yamazaki Gakuen University, 4-7-2 Minami-osawa, Hachioji, Tokyo 192-0364 Japan; 7 Institute for Biology and Mathematics of Dynamical Cell Processes (iBMath), The University of Tokyo, 3-8-1 Komaba, Tokyo, 153-8914 Japan; 8 Cardiovascular Research Institute, Present Address: University of California San Francisco, 555 Mission Bay Boulevard South, San Francisco, CA 94143 USA; 9 Department of Molecular Physiology, Present Address: National Cerebral and Cardiovascular Center Research Institute, 5-7-1 Fujishirodai, Suita, Osaka 565-8565 Japan; 10 Department of Cardiovascular Medicine, Present Address: Graduate School of Medical Sciences, Kumamoto University, 1-1-1 Honjo, Chuo-ku, Kumamoto 860-8556 Japan

## Abstract

The somatopleure is the amniotic primordium in amniote development, but its boundary to the embryonic body at early embryonic stages and the fate of cells constituting this structure are not well characterized. It also remains unclear how cells behave during the demarcation between intra- and extra-embryonic tissues. Here we identify cellular alignments, which indicate two streams towards the sites of dorsal amniotic closure and ventral thoracic wall formation. A subpopulation of mesodermal cells moving ventrally from the somatopleural region adjacent to the base of the head fold enter the body of the embryo and distribute to the thoracic wall, pharyngeal arches and heart. These cells are induced to differentiate into vascular endothelial cells and cardiomyocytes possibly by FGF and BMP signaling, respectively. These results indicate that the somatopleure acting as the amniotic primordium also serves as a source of embryonic cells, which may contribute to cardiovascular development.

## Introduction

In the history of vertebrate evolution, terrestrialization, the habitat transition from water to land, eventually lead to the prosperity of descendant species by expanding their living space, but the ancestral animals were subjected to harsh and threatening environments different from their native aquatic ones. Among various innovations to adapt to the aerial environment, the evolution of extraembryonic membranes including the amnion, chorion and allantois in the common ancestor of amniotes (reptiles, birds and mammals), which diverged from amphibians about 360 million years ago^1, 2^, contributed to the protection, respiration and nutrition of embryos and thereby successful reproduction^3, 4^.

Extraembryonic membranes, including the amnion, are formed as structures continuous with the embryonic tissues^5–7^. In chicken, the extraembryonic tissues are separated into two layers: the splanchnopleure composed of the endoderm and splanchnic mesoderm, and the somatopleure composed of the ectoderm and somatic mesoderm along with the formation of the coelomic cavity after gastrulation^5, 6^. The extraembryonic splanchnopleure gives rise to the yolk sac and allantois, whereas the somatopleure differentiates into the amnion and chorion with the fold of ectamnion as a boundary^5, 6^. The yolk sac, in addition to its primary role in nourishing the embryo, serves as the place of primary hematopoiesis to supply hematopoietic precursors to the embryo, while the amnion and chorion, which contribute to the respiration and protection of the embryo, are avascular, and no direct contribution as cell sources has been known^5^.

In chicken development, the embryonic/extraembryonic boundary is first defined morphologically at the early head-fold stage^6^. Although the boundary seems evident at the level of the head fold to the anterior intestinal portal, it is histologically ambiguous and not well characterized before. However, the somatopleure of this area outside the embryo proper is generally regarded as amniogenic, as evidenced by the separation of this area into the amnion and the chorion by the formation of the anterior and lateral amniotic folds^8^. In our present study, we examined the dynamics and fate of cells constituting this area (referred to as the “amniogenic” somatopleure thereafter) and identified streams of somatopleural cells to form the amnion. In addition, we found that a substantial cell population in the amniogenic somatopleural mesoderm enters the body of the embryo. In particular, somatopleural cells adjacent to the embryonic body at the level of the midbrain and anterior hindbrain migrate into the pharyngeal arches and distribute to the pharyngeal mesenchyme and the outflow tract of the heart as well as the thoracic wall, indicating that this somatopleural area may contribute to heart development as a novel cellular origin. Some cells migrating into the pharyngeal region are likely to be incorporated into the vascular network. These findings may reveal a novel role of the amniogenic somatopleure as a cellular source for embryonic development in amniotes.

## Results

### Fate analysis of the amniogenic somatopleure in chick embryos by fluorescent dye labeling

We applied DiI or CFDA/DiO lipophilic fluorescent dye to label amniogenic somatopleural cells at mid- to hindbrain levels in chick embryos at Hamburger and Hamilton stages (HH) 9 to 12– (6- to 15-somite stages). Among 413 dye-injected embryos, 150 (36.3%) embryos that developed normally without obvious malformations were subjected to fate-mapping analysis. The specificity of labeling was ensured by immunostaining sections of dye-labeled embryos. In the example of embryos labeled with DiI at 9ss, signals were solely detected in the cytokeratin-positive amniogenic somatopleure, without overlapping with Isl1 or Nkx2.5 staining in the embryonic mesoderm (Fig. S1).

**Figure 1:**
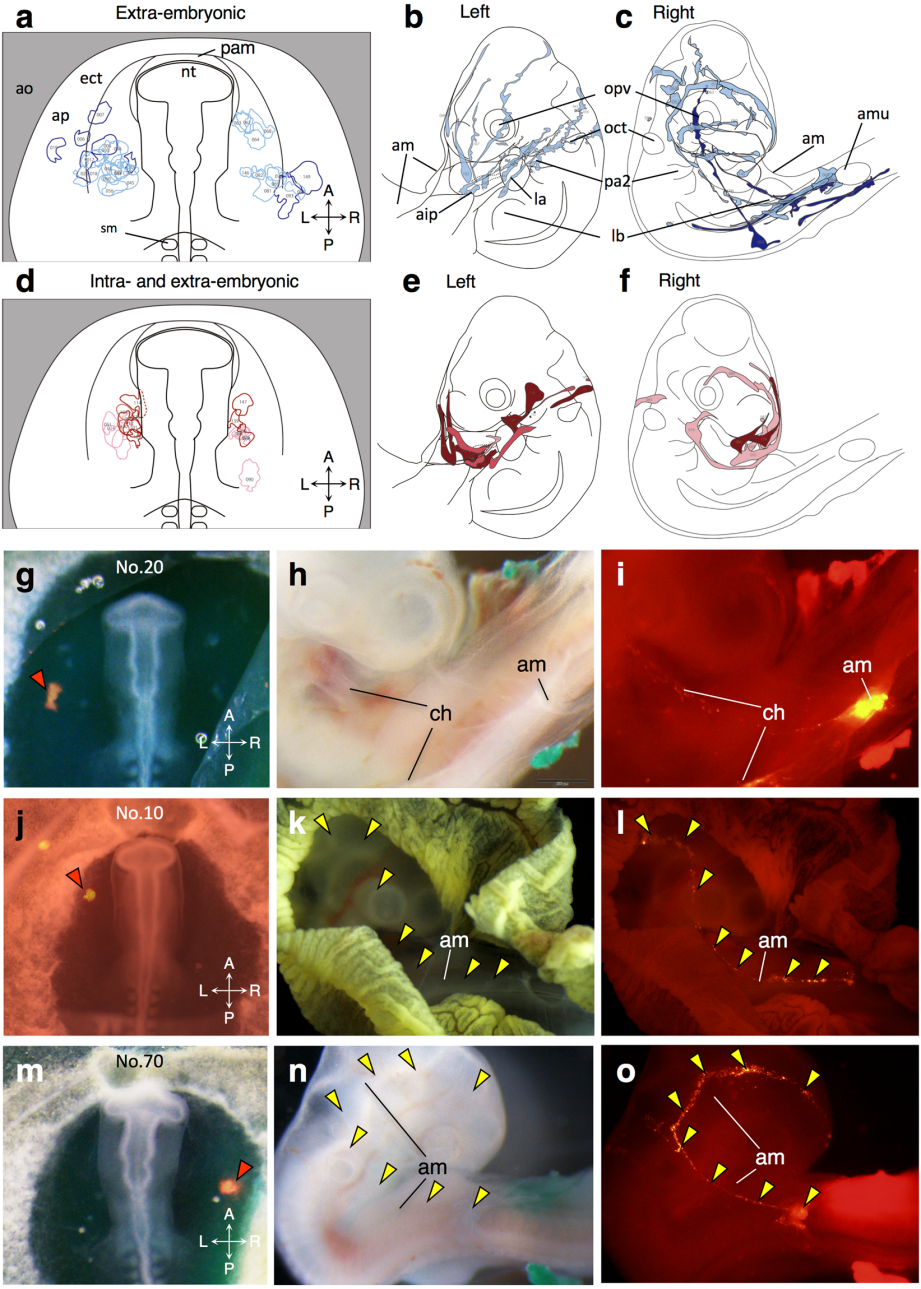
Fate analysis of the amniogenic somatopleure by fluorescent-dye injection. (**a**–**f**) Dye-injected sites in the amniogenic somatopleure at 9ss to 15ss (HH10– to HH12–) and distributions after 48 hours or longer incubation (equivalent to HH18 to 24) are plotted onto schematic illustrations with different colors as indicated in Fig. S2. A, anterior; P, posterior; L, left; R, right. (**g**–**o**) Contribution of amniogenic somatopleural cells to the amnion and chorion. Representative photos for DiI-injected sites (orange arrowheads in **g**,**j**,**m**) and later distributions (**h**,**k**,**n** in bright field; **i**,**l**,**o** in dark field) are shown. No. 20 (**g**–**i**) contributes to both the amnion and chorion. No. 10 (**j**–**l**) and No. 70 (**m**–**o**) contribute to the sero-amniotic connection (yellow arrowheads) from left and right sides, respectively. aip, anterior intestinal portal; am, amnion; amu, amniotic umbilicus; ao, area opaca; ap, area pellucida; ch, chorion; ect, ectamnion; la, left atrium; lb, limb bud; opv, optic vesicle; otv, otic vesicle; pam, proamnion; pa2, second pharyngeal arch; sm, somite.

Table S1 provides a detailed summary of dye-labeling experiments. Each sample is classified according to final location of dye-labeled cells and the labeled area is plotted onto schematic templates illustrating the anterior half of the embryo and adjacent area pellucida at equivalent stages (Fig. 1a,d; Figs S2 and S3). Distribution of fluorescent signals after 48 hours or longer incubation (HH18 to 24) was also mapped onto schematic templates (Fig. 1b,c,e,f).

In chick development, a horseshoe-shaped band of ectodermal thickening called “ectamnion” appears in the somatopleure from 9ss (HH10–), which has been regarded as a presumptive boundary between the inner the amniogenous and outer choriogenous somatopleure^9, 10^. In fact, somatopleural cells around and outside the ectamnion preferentially contributed to the chorion, although these cells also contributed to the amnion as all labeled-cells inside the ectamnion did (Fig. 1g–i; Fig. S4), justifying the term ‘amniogenic somatopleure’.

The ectamnion is assumed to develop as a crescentic ridge of the anterior amniotic fold and two arms of the lateral amniotic folds, which start to overlap the embryo from HH12. Then, the anterior and lateral amniotic folds unite in the midline with the later-formed posterior amniotic fold to form a closed sac of the amnion inside and a continuous membrane of chorion outside around HH18, leaving an attachment of the two membranes in the midline, the so-called sero-amniotic connection^5, 11^. During this process, the embryo undergoes cranial and cervical flexure with the head turned to the right. As a result, the sero-amniotic connection is located over the right side of the embryo towards the hindlimb. At HH18 and later, when dye-injected embryos were observed for the destination of labeled cells, many embryos showed an alignment of labeled cells along a trajectory towards the presumptive line of sero-amniotic connection (Fig. 1a–f; Fig. S4). Some embryos exhibited an alignment of labeled cells along the sero-amniotic connection itself (Fig. 1j–o). On the ventral side, the pericardial fold is formed as a boundary between the amnion and the anterior thoracic wall and descends from the pharyngeal arch region to the anterior intestinal portal beginning at HH17^12^. Correspondingly, collective mapping of labeled cells showed an array of alignments towards the anterior intestinal portal (Fig. 1a–e; Fig. S4). These distribution patterns indicate two distinct streams towards the dorsal and ventral closure sites of the amniotic and pericardial cavities, respectively. Notably, the proximal region inside the ectamnion at the level of the midbrain and hindbrain appeared to distribute ventrally, converging to the anterior intestinal portal.

### Intraembryonic contribution of the amniogenic somatopleure

**Figure 2:**
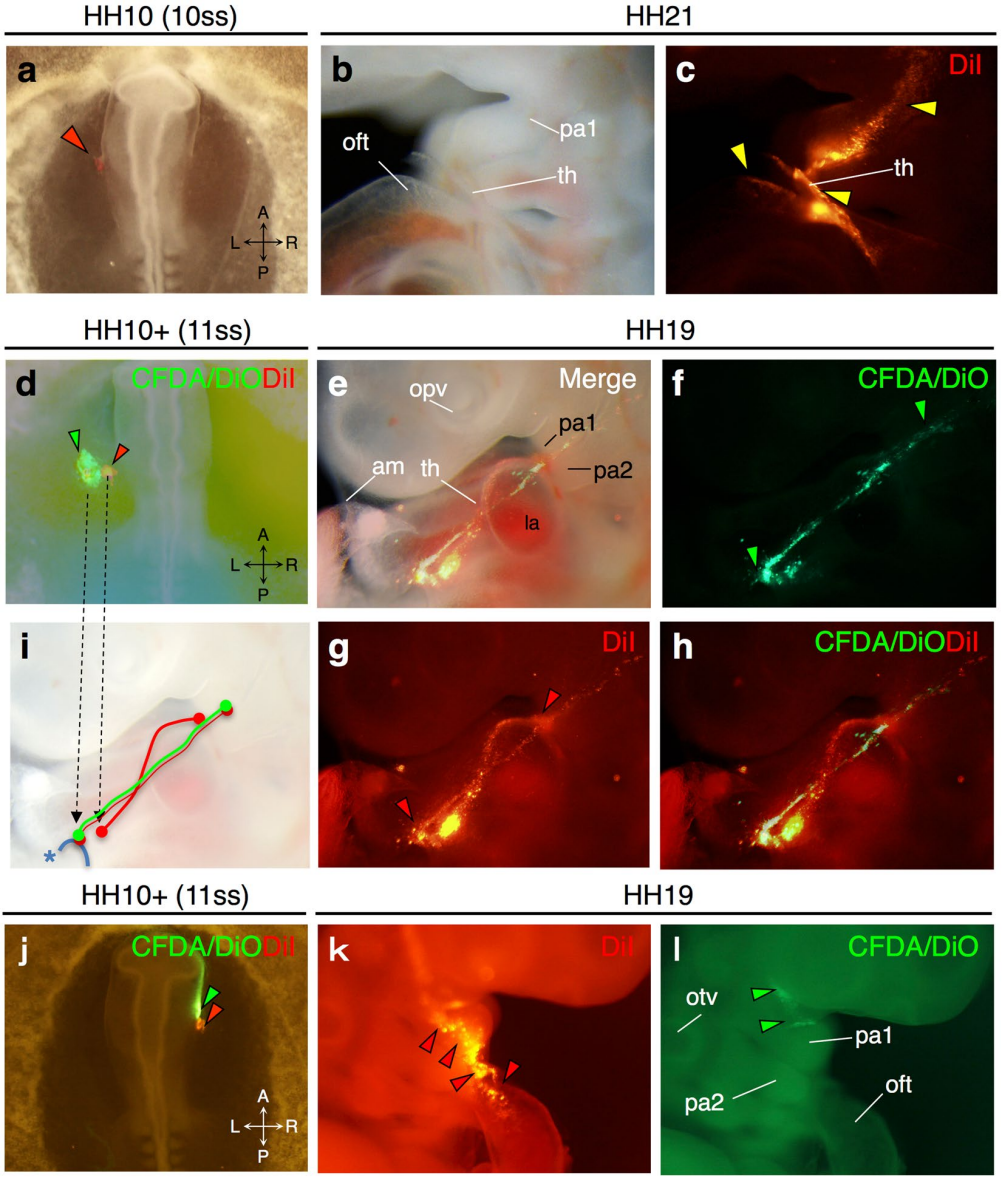
Behaviors of amniogenic somatopleural cells contributing to intraembryonic tissues. (**a**–**c**) Representative images of DiI-labeled somatopleural cell distribution to intraembryonic tissues including the thoracic wall, pharyngeal arch and cardiac outflow tract (yellow arrowheads). (**d**–**i**) Two-color dye labeling reveals a trajectory of somatopleural cells toward the pharyngeal arch through the pericardial fold. Both CFDA/DiO (green) and DiI (red) labels likely draw trajectories dorsoventrally on the amnion, whereas only DiI labels direct towards the ventral tip of the first pharyngeal arch on the forming thoracic wall (**i**). (**j**–**l**) Two-color dye labeling reveals that somatopleural cells and cells in the lateral edge of the head fold are likely to distribute differently with a boundary around the first pharyngeal arch. Dye-injected sites (red arrowheads in **a**,**d**,**j**) and later distributions (**b**,**e** in bright field; **c**,**f**–**h**,**k**,**l** in dark field) are shown. am, amnion; la, left atrium; lb, limb bud; opv, optic vesicle; otv, otic vesicle; pa1, first pharyngeal arch; pa2, second pharyngeal arch; th, thoracic wall; *, edge of the closing pericardial fold.

In addition to the contribution to the amnion and chorion, a population within the region converging to the anterior intestinal portal was found to contribute to the embryonic body (Fig. 1d–f; Figs S2–S4). Labeled cells in the most proximal region abutting on the base of the head fold were later detected not only in the amnion but also in the thoracic wall, the first or second pharyngeal arches and the heart at HH18 to HH21 (Figs S2–S4). Typically, fluorescent signals drew a trajectory towards the ventralmost portion of the first or second pharyngeal arches, where the signals diverged dorsally into the arch regions and ventrocaudally into the cardiac outflow tract (Fig. 2a–c). Although the contribution of labeled cells to the heart was mostly in the outflow tract, two embryos showed a contribution to the inflow tract close to the right atrium and the pericardium (Fig. S4). Some embryos showed an alignment of labeled cells along the thoracic wall midline, as seen in the sero-amniotic connection (Fig. S4). The contribution to intraembryonic tissues was getting smaller with lateral distance from the base of the head fold (Fig. 1d–f; Fig. S3), indicating that the position within the somatopleure relative to the base of the head fold is likely to determine their final destinations.

We also examined the relationship between the labeled site and final destination of labeled cells at HH9− to HH9+ (6ss to 8ss) (Fig. S5). The pattern of the regional specificity in the somatopleure was similar to that at HH10− to HH12−. These data suggest that the final destination of amniogenic somatopleural cells may be largely pre-determined at least by HH9+, before appearing the ectamnion.

To confirm the intraembryonic movement of extracellular somatopleural cells by different means, we performed transposon-mediated EGFP gene transfer at HH10- to HH10+ (9ss to 11ss) by electroporation (Fig. S6). Both ectodermal and mesodermal layers were assumed to be labeled by this method. After forty-eight hours, EGFP-labeled amniotic cells in the left lateral fold migrated into the tip of the pharyngeal arches and outflow tract, in consistence with the results in dye-labeling experiments (Fig. S6).

To further examine the regional specificity in the intraembryonic contribution of amniogenic somatopleural cells, we analyzed the distribution of fluorescent signals in embryos that underwent double dye labeling at different locations. When DiI (medially) and CFDA/DiO (laterally) were injected into the region beside the base of the head fold, the two fluorescent signals were overlapped in the same trajectory, but only DiI signals reached to the ventral tip of the first pharyngeal arch and entered the embryonic body (Fig. 2d–i). This result indicates that cells arising in the adjacent areas might take a common path towards the forming thoracic wall, but their final distributions might be distinct depending on the timing of incorporation into the pericardial fold. In another series of double labeling experiments, DiI and CFDA/DiO were separately injected into the amniogenic somatopleure just beside the base of the head fold and the adjacent lateral edge of the head fold on the right side. Among 25 embryos, 24 showed fluorescent signals applied to the amniogenic somatopleure later in the mandibular process of the first pharyngeal arch and/or the second pharyngeal arch, and 16 showed signals in the cardiac outflow tract, (Fig. 2j–l). By contrast, fluorescent signals applied to the lateral edge of the head fold were mostly detected in the maxillary process and, in some embryos, the oral surface of the mandibular process without overlapping with signals applied to the amniogenic somatopleure (Fig. 2j–l), indicating a demarcation of the intraembryonic region contributed by the amniogenic somatopleure with a boundary around the first pharyngeal arch.

### Characterization of the behavior of amniogenic somatopleural cells contributing to the intraembryonic tissues

**Figure 3:**
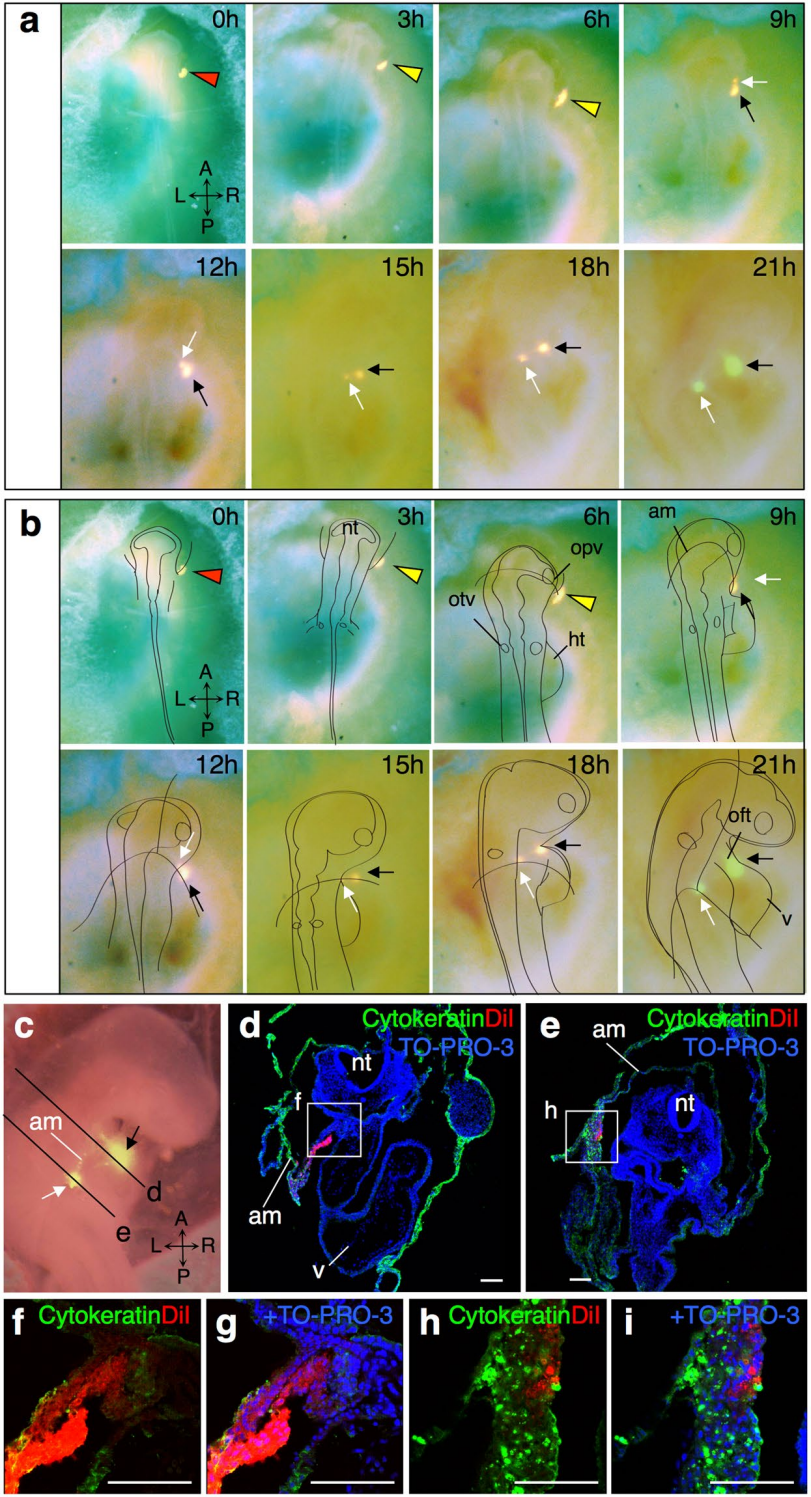
Sequential imaging of the dye-labeled cells in the amniogenic somatopleure forming the amniotic folds. (**a**) Dorsal view of sequential imaging of every 3 hours for 21 hours. DiI is injected in the amniogenic somatopleure in the right side of the head (red arrowhead). After 6 to 9 hours, the dye-labeled cells (yellow arrowheads) segregate into two groups; one is the mesodermal layer (black arrows) and the other is the amniotic membrane with the ectodermal epithelium (white arrows). (**b**) Schematic depiction is superimposed on each image. (**c**–**g**) Right lateral view (**c**) and immunostaining for the epithelial cell marker cytokeratin on transverse sections (**d**–**i**) of the embryo shown in (**a**). Boxes in (**d**,**e**) are magnified in (**f,g**) and (**h,i**) respectively. am, amnion; ht, heart tube; nt, neural tube; oft, outflow tract; opv, optic vesicle; v, ventricle. Scale bars, 300 µm for (**d**) to (**i**).

To analyze the dynamics of amniogenic somatopleural cells leading to distinct distributions, we placed relatively broad labels onto the area beside the base of the head fold and observed changes in their distributions. Labeled cells were segregated into two populations around 6 to 9 hours after labeling: thereafter one moved dorsally and integrated into the sero-amniotic connection, whereas the other moved ventromedially towards the forming thoracic wall (Fig. 3a,b). Immunohistochemical staining of sections after 21 hour incubation revealed that the former cell population was incorporated into a cytokeratin-positive cell cluster corresponding to the sero-amniotic connection, whereas the latter cell population was detected in the mesodermal layer underlying the thoracic wall close to the root of the aortic sac at the ventral region of the second pharyngeal arch (Fig. 3c–i). These data suggest that the cell population contributing to the intraembryonic tissues are mainly from the mesodermal layer and move differently from the cells contributing to the amnion. Furthermore, these cells move towards the ventral region of the pharyngeal arch before the beginning of pericardial formation at HH16.

### Amniogenic somatopleural cells differentiate into vascular endothelial cells and cardiomyocytes

**Figure 4:**
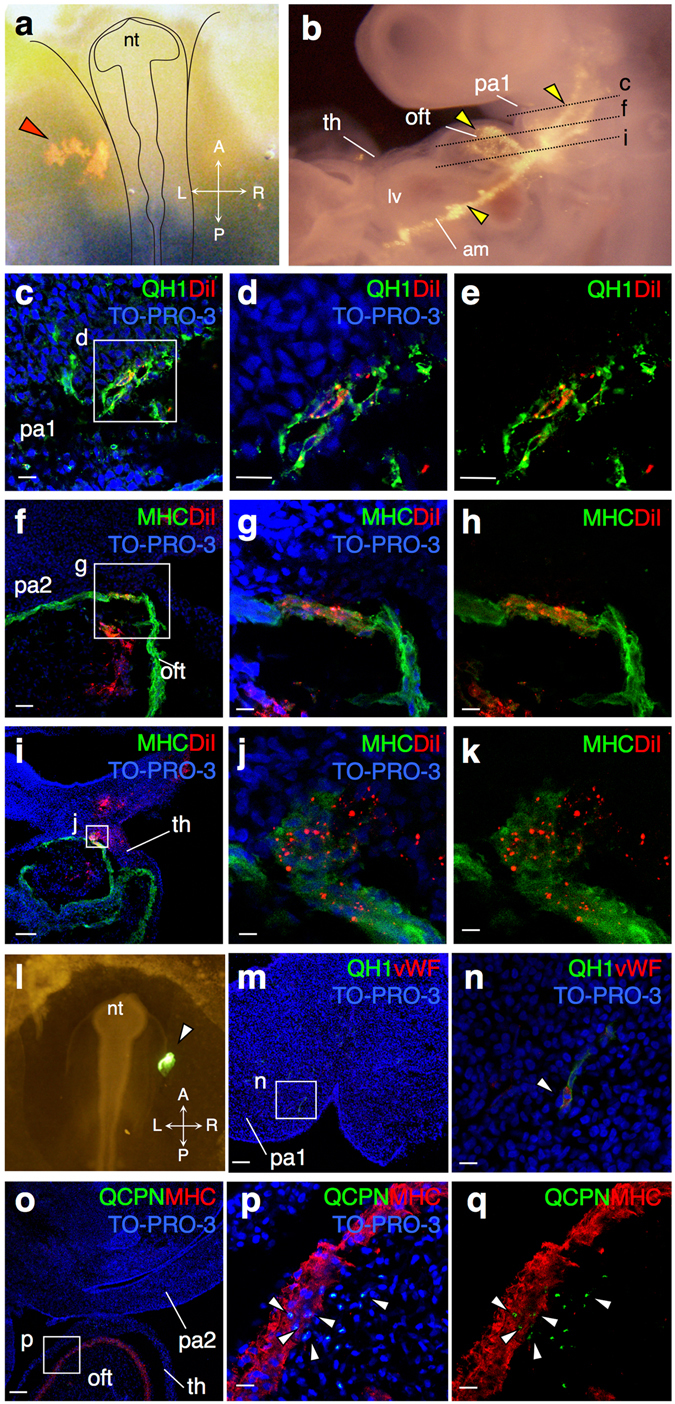
Amniogenic somatopleural cells differentiate into vascular endothelial cells and cardiomyocytes. (**a**–**k**) Dye-labeling in quail embryos. DiI is injected onto the amniogenic somatopleure adjacent to the head fold in a quail embryo at 10ss (red arrowhead) (**a**). Schematic depiction is superimposed. (**b**) Forty-eight hours after DiI injection (equivalent to HH18+), dye-labeled cells distribute to the amnion, the first pharyngeal arch, the outflow tract and pericardium. Yellow arrowheads indicate distribution of DiI in the embryonic tissues. (**c**–**k**) Immunostaining of transverse sections of the embryo shown in (**b**) at different levels. Boxes in (**c**,**f**,**i**) are magnified in (**d**, **e**), (**g**, **h**) and (**j**, **k**), respectively. DiI-labeled cells are detected in the QH1-positive vascular endothelium in the first pharyngeal arch (**c**–**e**) and myosin heavy chain (MHC)-positive myocardium in the outflow tract (**f**–**k**). (**l**–**q**) Quail-chick chimera experiments. A quail amniogenic somatopleure graft is orthotopically transplanted into the chick somatopleure at 10ss (**l**). The graft is visualized by DiO-labeling (white arrowhead). (**m**–**q**) Immunostaining of transverse sections of the recipient embryo after 48 hours. Boxes in (**m**,**o**) are magnified in (**n**) and (**p**, **q**), respectively. QH1- or QCPN-positive quail cells (white arrowheads) are detected in the von Willebrand factor (vWF)-positive vascular endothelium in the first pharyngeal arch (**m**,**n**) and MHC-positive myocardium in the outflow tract (**o**–**q**). TO-PRO-3 is used to counterstain nuclei. nt, neural tube; oft, outflow tract; pa1, first pharyngeal arch; pa2, second pharyngeal arch; th, thoracic wall. Scale bars, 100 µm for (**f**,**i**,**m**,**p**), 20 µm for others.

To investigate the fate of amniogenic somatopleural cells entering the embryo, we examined the distribution and characteristics of cells labeled with fluorescent dyes by immunohistological analysis using quail embryos at 10ss (Fig. 4a). At the stage equivalent to HH18+, the labeled cells were distributed in the pharyngeal mesenchyme, cardiac outflow tract and thoracic wall in quail embryos (Fig. 4b–k). The distribution patterns in quail embryos were almost the same as in HH18+ chick embryos (Figs S1a,f and S4). In the pharyngeal region, some labeled cells were incorporated into vessel walls, which were proved to be vascular endothelial cells by positive staining for the quail endothelial marker QH1 (Fig. 4c–e). In the cardiac outflow tract, labeled cells were distributed into different layers, including the endocardium, myocardium and cushion tissues (Fig. 4f–k). In the myocardial layer, the labeled cells were positive for the cardiomyocyte marker myosin heavy chain (MHC) (Fig. 4f–k), indicating the direct contribution of the amniogenic somatopleure to cardiomyocytes. Labeled cells were also detected in the midline of the thoracic wall (Fig. 4b).

To follow the final destinations of labeled cells in the mature heart, we examined their distribution and characteristics in quail embryos at HH35 (Day 8.5) (Fig. S7). These cells distributed in the midline of the body wall, the aorta, and the heart (Fig. S7), as observed at HH18+ (Fig. 4b). The fluorescent signals were detected in the myocardial layer close to the aortic root, overlapping with MHC staining (Fig. S7). These results suggest that amniogenic somatopleural cells differentiate into various cell types including vascular endothelial cells and cardiomyocytes and contribute to the mature heart and vessels.

To confirm the results from dye-labeling experiments, we performed quail-chick chimera transplantation. The amniogenic somatopleure between the embryonic body and ectamnion at the level of the hindbrain excised from quail embryos at HH10 were orthotopically grafted into the chick embryos at the same stage. After 48 hours, QH1-positive quail cells were found in the vascular endothelium and surrounding mesenchyme in the pharyngeal arches (Fig. 4l–q). QH1-positive cells were also stained for von Willebrand factor, another specific marker of vascular endothelial cells, confirming the endothelial differentiation of transplanted quail cells (Fig. 4m,n). Quail cells, identified with the quail-specific QCPN antibody, were also found in the myocardial layer of the cardiac outflow tract, cushion tissue and pericardium (Fig. 4o–q). These QCPN-positive cells, derived from a quail-graft, were identified as MHC-positive cardiomyocytes (Fig. 4o–q).

### FGF and BMP signals are involved in endothelial and myocardial differentiation of amniogenic somatic mesodermal cells

Before the beginning of pericardial formation at HH16, the amniogenic somatopleure is continuous to the root of the aortic sac at the ventral region of the second pharyngeal arch, where cells are likely to enter the embryonic body towards the pharyngeal region and cardiac outflow tract (Fig. S8a–c). To investigate which signals around this putative portal site might be involved in the multi-lineage differentiation of the amniogenic somatic mesoderm, we performed *in situ* hybridization on chick embryo sections at the level of the pharyngeal region and cardiac outflow tract. It has been well known that FGF and BMP signals are involved in angiogenesis and cardiac development by regulating the differentiation of undifferentiated progenitors into vascular endothelial cells and cardiomyocytes^13, 14^. In line with previous work^15^, we detected local expression of *Fgf8* in the pharyngeal endoderm and surface ectoderm, and *Bmp2* and *Bmp4* in the pharyngeal mesoderm and endoderm and the root of the cardiac outflow tract, where migrating somatic mesodermal cells pass into the embryonic body at comparable developmental stages (Fig. S8d–i).

**Figure 5:**
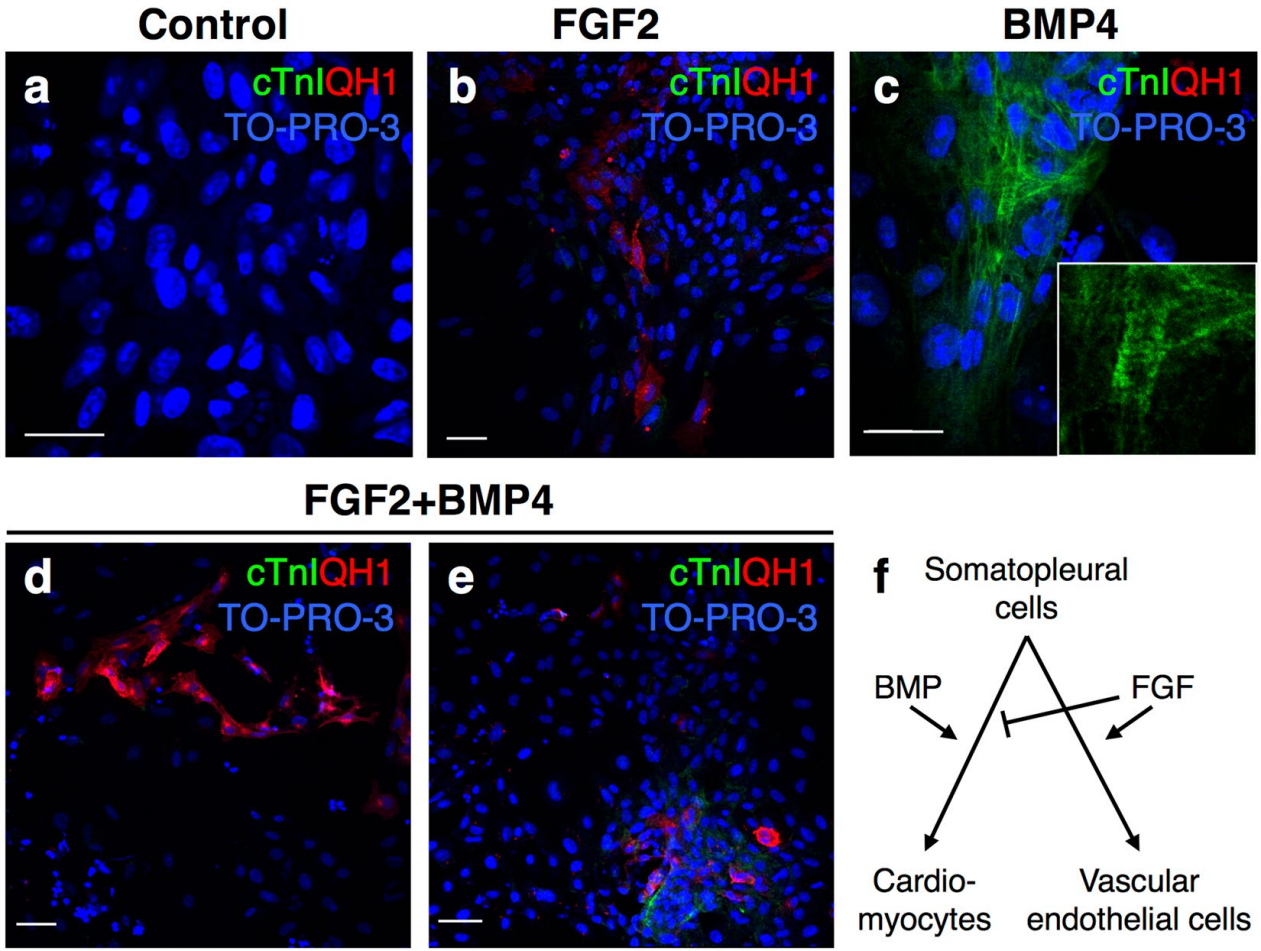
FGF and BMP signaling induce vascular endothelial cell and cardiomyocyte differentiation, respectively, in amniogenic somatopleure explants. (**a**–**e**) Amniogenic somatopleure explants from quail embryos at 9ss to 11ss are cultured in the presence of FGF2 (10 ng/ml) (n = 13) (**b**), BMP4 (300 ng/ml) (n = 14) (**c**) or both (n = 12) (**d**,**e**) and immunostained for the cardiomyocyte marker cardiac troponin I (TnI) and quail-specific vascular endothelial cell marker QH1. Control explants (n = 11) show staining for neither marker (**a**). Stimulation with FGF2 or BMP4 induces differentiation of quail somatopleural cells into vascular endothelial cells and cardiomyocytes, respectively (**b**,**c**). Simultaneous stimulation with FGF2 and BMP4 results in only vascular endothelial differentiation (**d**) or differentiation into both endothelial cells and cardiomyocytes (**e**) in 8 and 4 of 12 explants, respectively. TO-PRO-3 is used to counterstain nuclei. (**f**) Summary of the effects of FGF and BMP signaling on somatopleural cell differentiation. Scale bars, 15 µm for (**a**,**c**) and 40 µm for (**b**,**d**,**e**).

**Figure 6:**
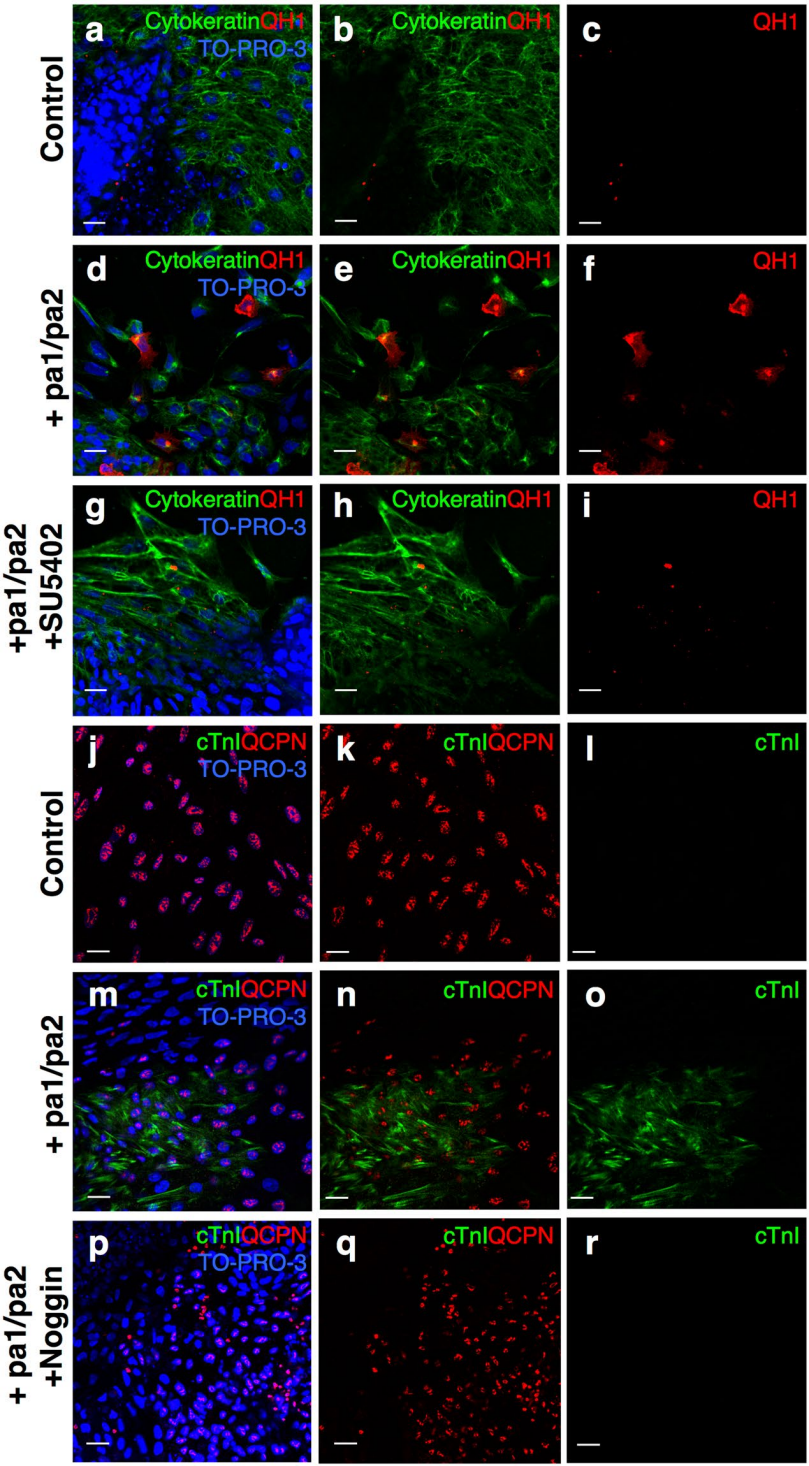
Pharyngeal arch tissues induce differentiation of amniogenic somatopleural cells into vascular endothelial cells and cardiomyocytes, possibly via FGF and BMP signaling, respectively. (**a**–**r**) Amniogenic somatopleural explants from quail embryos at 9ss to 11ss are co-cultured with chick first and second pharyngeal arch (pa1/pa2) explants at HH15 and immunostained for QH1 and cytokeratin (**a**–**i**) or QCPN and cardiomyocyte marker cardiac troponin I (cTnI) (**j**–**r**). (**a**–**i**) Quail somatopleural explants alone do not show QH1-positive endothelial cells (n = 7) (**a**–**c**). By contrast, co-culture with pharyngeal arch explants induces endothelial cell differentiation (n = 7) (**d**–**f**), which is inhibited by FGF receptor antagonist SU5402 (20 µM) (n = 7) (**g**–**i**). (**j**–**r**) Although quail somatopleural explants alone do not show cTnI-positive cardiomyocytes (n = 3) (**j**–**l**), co-culture with pharyngeal arch explants induces cardiomyocyte differentiation (n = 3) (**m**–**o**), which is inhibited by Noggin (500 ng/ml), a BMP antagonist (n = 4) (**p**–**q**). TO-PRO-3 is used to counterstain nuclei. Scale bars, 20 µm.

Then we cultured quail amniogenic somatopleure explants and examined the effects of these signaling molecules. Under basal condition, almost all the cultured cells derived from the explants were positive for cytokeratin, but QH1 signals were hardly detected (Fig. S9). Stimulation of FGF signaling with FGF2 highly induced QH1 expression, indicating the differentiation into endothelial cells (Fig. 5a,b; Fig. S9), whereas BMP signaling stimulation did not induce QH1 expression (Fig. 5c; Fig. S9). The induction of QH1 in amniogenic somatopleure-derived cells was downregulated by the FGF receptor antagonist SU5402 (Fig. S10). Furthermore, co-culture of somatopleure explants with chick pharyngeal arch explants caused the induction of QH1, which was abolished by SU5402 (Fig. 6a–i). These results indicate that the pharyngeal arch tissues can induce endothelial differentiation of somatopleure-derived cells through FGF signaling.

In terms of cardiomyocyte differentiation, BMP signaling stimulation with BMP4 induced expression of cardiac troponin I (cTnI) and Nkx2.5 in the amniogenic somatopleure explants, indicating differentiation into cardiomyocytes (Fig. 5a,c; Fig. S11). This induction was inhibited by the BMP extra-cellular antagonist Noggin (Fig. S11). Moreover, co-culture of somatopleure explants with chick pharyngeal arch explants also caused the induction of cTnI and Nkx2.5 expression, which was suppressed by Noggin (Fig. 6j–r; Fig. S12). These results indicate that the pharyngeal arch tissues can induce cardiomyocyte differentiation of the somatopleure-derived cells through BMP signaling.

Finally, we examined possible interaction between FGF and BMP signaling by exposing amniogenic somatopleure explants simultaneously to both FGF2 and BMP4 (Fig. 5d,e). In 33.3% of explants (4/12), we detected both QH-1-positive endothelial cells and cTnI-positive cardiomyocytes, whereas the other 66.7% (8/12) showed only QH1-positive cells (Fig. 5d,e). This result suggests that FGF signaling may overwhelm the effect of BMP, resulting in a tendency towards vascular endothelial differentiation of somatopleure-derived cells (Fig. 5f).

## Discussion

In the present study, we investigated the dynamics and fate of amniogenic somatopleural cells and identified a pattern of alignment in both the dorsal and ventral directions to form the amniotic sac. A subpopulation of amniogenic somatopleural cells moving ventrally forms the pericardial fold and enters the embryonic body. These cells display mesodermal characteristics and differentiate into various cell types including vascular endothelial cells and cardiomyocytes, possibly involving FGF and BMP signaling.

### Cellular dynamics underlying amnion formation

**Figure 7:**
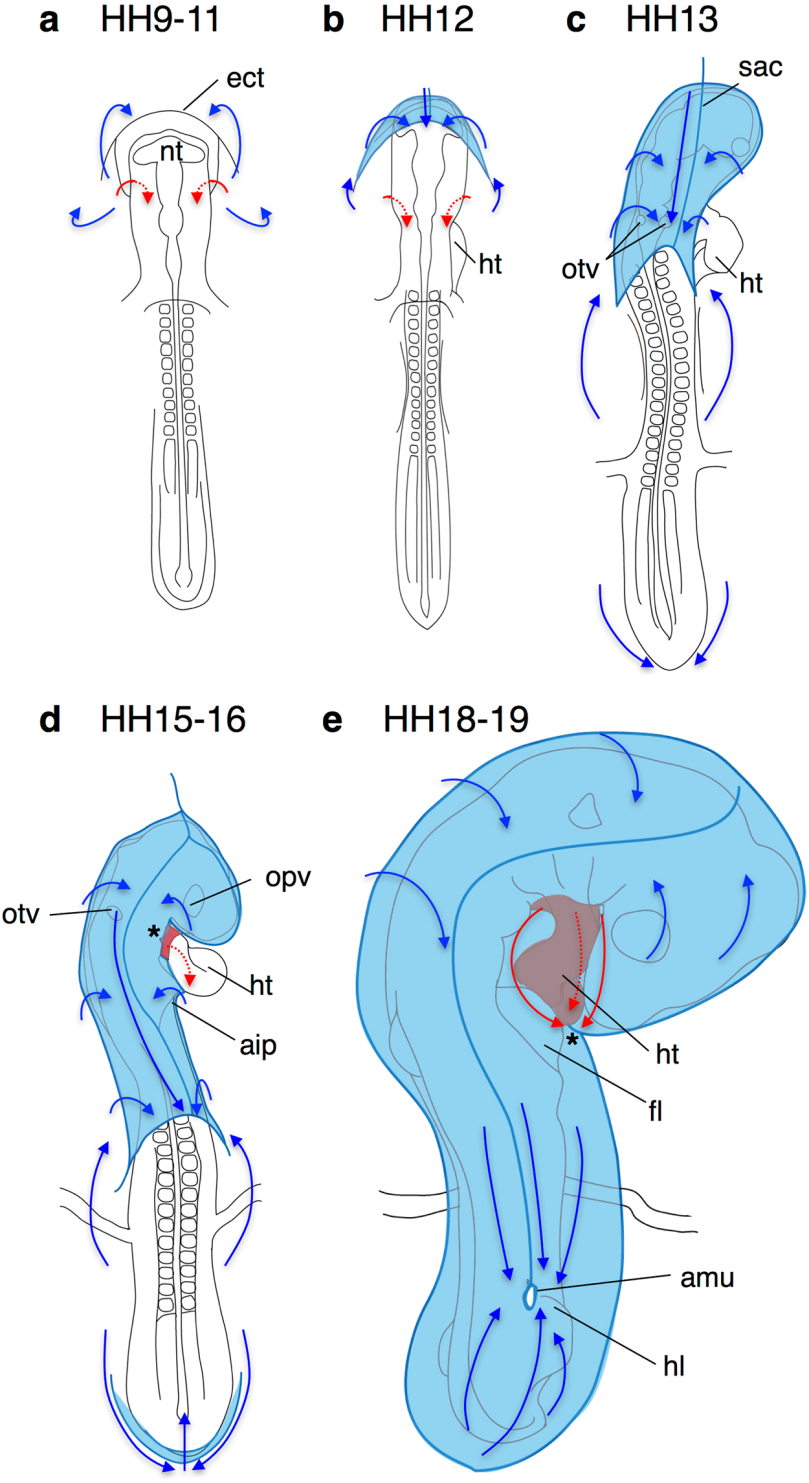
Presumptive streams of amniogenic somatopleural cell movement forming the amnion and entering the embryonic body. Schemas indicate a dorsal view of amnion-forming embryos. Red arrows indicate direction of the ventrad movement to close the pericardial cavity; blue arrows indicate direction of the dorsad movement to close the amnion. Asterisks in (**d**,**e**) indicate the edge of the pericardial fold. aip, anterior intestinal portal; am, amnion; amu, amniotic umbilicus; fl, forelimb; hl, hindlimb; ht, heart tube; opv, optic vesicle; otv, otic vesicle; sac, sero-amniotic connection.

The amnion and chorion are derived from the somatopleure with a presumptive border of the ectamnion. Following the anterior extension of the extraembryonic mesoderm and formation of the coelom, the anterior and lateral amniotic folds arise along the ectamnion and grow posteriorly over the head of the embryo^8^. These folds meet the posterior amniotic fold that develops covering the tail region of the embryo to fuse together above the right hindlimb. The dorsad cellular stream indicated by labeled cell distribution seems to correspond to this movement towards the amniotic closure site. On the other hand, the ventral thoracic wall is formed by the descent of the pericardial fold from the pharyngeal arch region to the anterior intestinal port. The ventrad stream of amniogenic somatopleural cells may correspond to this descending movement of the pericardial fold. These presumptive cell movements to form the amniotic and pericardial cavities are summarized as a series of schematic drawings in Fig. 7.

A recent report has demonstrated cellular dynamics during avian and crocodile amniotic formation, which shows that the anterior and lateral amniotic folds form along a stiff cellular ring by buckling events due to contrast of elasticity^16^. Around the time of amnion folding, cells are aligned circumferentially along the edge of the folds, but perpendicularly in the surrounding amniotic area away from the edge, where the tissue is stretched radially. The edge of the folds thereafter constricts posteriorly like a purse string under the radial stretching force to close the amniotic sac. The present results are consistent with this dynamics, indicating that amniogenic somatopleural cells proliferate and align radially along the direction of stretching force, appearing as the dorsad stream. The similar distribution pattern of the ventral region suggests that the dynamics of thoracic wall formation may share similar characteristics with that of amniotic formation with the pericardial fold as a dividing ridge between extraembryonic and intraembryonic cellular contribution.

### Contribution of the amniogenic somatopleure to heart and vessel formation

The present study has further identified a portion of the amniogenic somatopleure adjacent to the base of the head fold as the region contributing to embryonic tissues in the thoracic wall and pharyngeal and cardiac regions. The somatopleure is known to serve as the matrix of the ventrolateral body wall and gives rise to connective tissue, tendons and the sternum^17–19^. In this regard, it is remarkable that some embryos showed alignment of labeled cells along the ventral midline, which might correspond to the primordium of the sternum. Furthermore, mesodermal cells from this region migrate into the embryonic body through the ventral region of the pharyngeal arches and differentiate into vascular endothelial cells and cardiomyocytes.

In chick development, vascular endothelial cells and cardiomyocytes normally derive from the splanchnopleural and paraxial mesoderm^20, 21^. By contrast, the somatopleural mesoderm does not contribute to these cell populations. Indeed, the amnion is formed as an avascular tissue, and the fate of amniogenic mesodermal cells seems to be quite restricted. However, previous reports have shown their differentiation potentials. In particular, there exists multipotent stem-like cell population in the amnion, which can differentiate to various cell types including cardiomyocytes^22–24^. The present study indicates that, after entering the embryonic body through the pharyngeal region, amniogenic somatopleural cells may escape from their fate restriction and exert their differentiation potentials in response to signaling cues from surrounding tissues.

At the entrance from the amniogenic somatopleure, some signaling molecules including FGFs and BMPs are expressed. Migratory cells might first receive FGF signals from the ectoderm and endoderm, and then a subpopulation of these cells may be differentiated into vascular endothelial cells to participate in the regional angiogenesis. Cells migrated further into the cardiac outflow tract subsequently receive BMP signals and their fates might be switched differently, partly to cardiomyocytes. BMP molecules have previously been reported to serve as a ectodermal signal to maintain the characteristics of the somatic mesoderm^25^. Thus, the same signal may be used for different fate determination before and after entering the embryonic body. Previously, it has been reported that a brief exposure to FGF largely enhances the cardiogenic activity of BMP in non-precardiac mesoderm derived from the posterior lateral plate of chick HH6 embryos^26, 27^. The present study, however, shows that FGF signaling may overwhelm the effect of BMP when they are applied simultaneously. Taken together, the signaling relay involving FGFs and BMPs may be critical in myocardial commitment and differentiation of the amniogenic somatopleural mesoderm during the normal developmental process in chicken.

The cardiovascular system now proved to be established through the assembly and interactions of various cell types of different origins^28–30^. In chick development, cardiac precursor cells migrate into the anterior lateral plate at the gastrula stage^31^. Following the split of the lateral plate into the somatopleure and splanchnopleure by coelom formation, cardiac precursors locate in the splanchnic mesodermal layer as the bilateral primary heart fields, which meet in the midline to form the primary heart tube^20^. Later, a subpopulation of the cranial paraxial mesoderm and the pharyngeal mesoderm serves as the second heart field to contribute to the outflow tract and right ventricle^20^. In addition, a caudal discrete region of the splanchnic mesoderm was recently identified as the tertiary heart field that gives rise to pacemaker cells^32^. The present study adds the somatopleural mesoderm as a novel origin of cardiomyocytes and suggests a certain population of somatopleural mesodermal cells may retain their potency to differentiate into cardiomyocytes after splitting from splanchnic cardiogenic mesoderm. The behavior of amniogenic somatic cells is somewhat similar to that of cells from the second heart filed in that they distribute to the pharyngeal mesenchyme and the outflow tract and differentiate into cardiomyocytes. Thus, amniogenic somatopleural cells may join to the embryonic paraxial and splanchnic mesoderm here and constitute a heterogeneous mesodermal population.

In vertebrate evolution, the amniotes developed four-chambered complex structures of the heart to accommodate themselves to a terrestrial life. Although it requires further studies to clarify whether cells of the somatopleure origin have a specific role in cardiovascular development, whether the cellular interaction involving amniogenic somatopleural cells in the pharyngeal region might contribute to the complexity and refinement of the amniote hearts is another interesting issue to pursue in future research.

## Methods

### Animals

Fertilized Hypeco Nera (Gallus gallus) eggs and Japanese quail (Coturnix japonica) eggs were obtained from Shiroyama Egg Farm (Kanagawa, Japan) and Motoki Hatchery (Saitama, Japan), independently, and were incubated at 37 °C in humid incubator at 37 °C to appropriate embryonic stages. All experiments using animals were reviewed and approved by Tokyo Women’s Medical University and the University of Tokyo Animal Care and Use Committee and were performed in accordance with the institutional guidelines for care and use of laboratory animals.

### *In situ* hybridization

Cryosection *in situ* hybridization was performed as previously described methods with minor modification^33, 34^. The *Fgf8* and *Bmp4* probes were kindly gifted from Dr. Mikawa. The *Bmp2* probes were from Dr. Ishii.

### Immunohistochemistry

Sections (14 μm) were prepared from frozen embryos, and were immunostained using the following antibodies: mouse monoclonal anti-quail cell (QCPN; Developmental Studies Hybridoma Bank; 1:100), mouse monoclonal anti-QH1 (Developmental Studies Hybridoma Bank; 1:100), mouse anti-Isl1 (Developmental Studies Hybridoma Bank; 1:100), mouse anti-MHC (MF20; Developmental Studies Hybridoma Bank; 1:100), mouse anti-sarcomeric α actinin (ab9465; Abcam; 1:100), rabbit anti-MHC (ab124205; Abcam; 1:100), rabbit anti-cardiac troponin I antibody (ab47003; Abcam; 1:100), rabbit anti-Nkx2.5 (ab35842; Abcam; 1:100). Signals were visualized with FITC (fluorescein isothiocyanate)- or TRITC-conjugated secondary antibodies specific for the appropriate species. Some sections were treated with biotin-conjugated secondary antibodies and visualized using the Cy3 streptavidin (Biolegend; 1:1000) or Dylight 488 streptavidin (Biolegend; 1:500). Nuclei were visualized with TO-PRO-3 Iodide (Molecular Probes; 1:1000). Fluorescent signals were visualized with a computer-assisted confocal microscope (Nikon ECLIPSE C2/Ti) and software (Nikon NIS-Elements AR4.100).

### Chick and quail embryos fluorescent dye injection

Fluorescent dye injection was performed as described previously^33, 35^ on 8-ss to 13-ss chick- or quail embryos using micropipettes. CM-DiI (Molecular Probes) and CFDA/DiO mixture (1:1; Molecular Probes) in DMSO (Wako) or N,N-dimethylformamide (Wako) was diluted in tetraglycol (Sigma) to a final concentration of 1 mg/ml for each. Embryo were visualized with Fast Green FDF (Wako) diluted 1:1000 in Tyrode’s solution, injected into the subgerminal cavity.

### Sequential imaging of chick embryo in ovo

CM-DiI labeled-quail embryos were prepared by the above-described dye injection method. Images were taken every 3 hours by using DP80 system (Olympus).

### Quail-chick amnion chimera

Quail-chick chimeras have been described previously^35, 36^. After incubation in a humid chamber at 37 °C, the fertilized quail and chicken eggs were opened in the shell. Stages of host chicken and donor quail embryos were match as closely as possible. Dissecting amnions from quail embryos were transplanted into the slit of chick amnion in head sides using fine grass needles.

### *In-vitro* cell culture

The amnions were excised from 8-ss to 12-ss of quail embryos, and placed in a collagen-coated glass-bottom 3.5-cm dish containing 100 µl DMEM (Wako)/M199 (Invitrogen) mixed medium (1:4) at 37 °C in 5% CO_2_ balanced with N_2_ for 24 hours or 48 hours. In organ culture, the first and second pharyngeal arches from chick embryos in 7- to 12-ss were collected, and co-culture with the quail amnion. In some experiments, mouse BMP4 (300 ng/ml, R&D), Noggin (500 ng/ml, R&D), rm-bFGF (FGF2) (10 ng/ml, R&D), SU5402 (20 µM, Wako) were added to the culture medium. The concentration of each agent was determined after preliminary experiments based on previous reports^37, 38^.

### Electroporation

DNA mixture (1.5 μg of the transposase expression plasmid, 1.5 μg of Tol2-GFP plasmid^39, 40^, 0.1% Fast Green and 1% sucrose diluted with Tyrode’s solution) was injected in the space between the left side of the head and the amnion with micropipettes. Cells were transfected by electroporation (5 successive square pulses of 25 V and 50 ms) with a pair of platinum wire electrodes and the electroporator (CUY21EDIT II, BEX). After 7 hours, the amniotic cells labeled with strong GFP fluorescence. The Tol2 system plasmids were gifted from Dr. Takahashi and Dr. Kawakami.

## Electronic supplementary material

Supplementary information
