# Supplementary material for: Amniogenic somatopleure: a novel origin of multiple cell lineages contributing to the cardiovascular system
Source: Sci Rep. 2017 Aug 21;7:8955. doi: 10.1038/s41598-017-08305-2 (PMC5566219; doi:10.1038/s41598-017-08305-2)
Supplement: Supplementary file 1 — Supplementary information [file 41598_2017_8305_MOESM1_ESM.pdf]

Supplementary information to:

Amniogenic somatopleure: a novel origin of multiple cell lineages contributing to the cardiovascular system

Rieko Asai[1,2,3,8], Yuka Haneda[1,2,4], Daiki Seya[1,2,9], Yuichiro Arima[1,10], Kimiko Fukuda[5], Yukiko Kurihara[1,2], Sachiko Miyagawa-Tomita[1,3,6,*] and Hiroki Kurihara[1,2,7,*]

[1]Department of Physiological Chemistry and Metabolism, Graduate School of Medicine, The University of Tokyo, 7-3-1 Hongo, Bunkyo-ku, Tokyo, 113-0033, Japan.
[2]Core Research for Evolutional Science and Technology (CREST), Japan Science and Technology Agency (JST), Chiyoda-ku, Tokyo, 102-0076, Japan.
[3]Department of Pediatric Cardiology, Tokyo Women's Medical University, 8-1 Kawada-cho, Shinjuku-ku, Tokyo 162-8666, Japan.
[4]Department of Biological Sciences, Graduate School of Science, The University of Tokyo, 7-3-1 Hongo, Bunkyo-ku, Tokyo 113-0033, Japan
[5]Department of Biological Science, Tokyo Metropolitan University, 1-1 Minami-osawa, Hachioji, Tokyo 192-0397, Japan.
[6]Department of Veterinary Technology, Yamazaki Gakuen University, 4-7-2 Minami-osawa, Hachioji, Tokyo 192-0364, Japan.
[7]Institute for Biology and Mathematics of Dynamical Cell Processes (iBMath), The University of Tokyo, 3-8-1 Komaba, Tokyo 153-8914, Japan.
[8]Present address: Cardiovascular Research Institute, University of California San Francisco, 555 Mission Bay Boulevard South, San Francisco, CA 94143, USA.
[9]Present address: Department of Molecular Physiology, National Cerebral and Cardiovascular Center Research Institute, 5-7-1 Fujishirodai, Suita, Osaka 565-8565, Japan.
[10]Present address: Department of Cardiovascular Medicine, Graduate School of Medical Sciences, Kumamoto University, 1-1-1 Honjo, Chuo-ku, Kumamoto 860-8556, Japan.

[*]Correspondence and requests for materials should be addressed to S.M.-T. (s_tomita@yamazaki.ac.jp) or H.K. (kuri-tky@umin.net)

Supplementary figure legends

Figure S1. Verification of fluorescent-dye labeling onto the amniogenic somatopleural ectoderm and mesoderm. (**a**) Dorsal view of a DiI-labeled site (red arrowhead) in the amniogenic somatopleure adjacent to the head fold in a chick embryo at 9ss (HH10–). The white dashed line indicates the level of transverse sections in **b** to **n**. (**b**) Hematoxylin-eosin staining of a transverse section of a chick embryo at 9ss. (**c**-**n**) Immunostaining of transverse sections indicated in A with antibodies for cytokeratin (an epithelial cell marker), Isl1 and Nkx2.5 (cardiac progenitor cell markers), superimposed with DiI signals. Boxes in **c**, **g, k** are magnified in **d**-**f**, **h**-**j** and **l**-**n**, respectively. TO-PRO-3 is used to counterstain nuclei. A, anterior; P, posterior; L, left; R, right; ec, ectoderm; iec, intra-embryonic coelom; lsm, left somatopleure; lsp, left splanchnopleure; nc, notochord; mes, mesoderm; nt, neural tube; pam, proamion; px, pharynx. Scale bars, 0.5mm for A, 100μm for others.

Figure S2. Mapping of injected areas in the amniogenic somatopleure. (**a**, **b**) Dorsal view of a chick embryo at 9ss with low (**a**) and high (**b**) magnifications. The schematic template used throughout this work is superimposed in **b**. (**c**) Dye-injected sites at 9ss to 15ss (HH10– to HH12–) plotted onto the schematic template. A total of 139 labeled sites are plotted with different colors according to their intra- and extra-embryonic contributions. Location of each site at different stages is determined relative to marker structures including divisions of the neuromeric structure, the first somite, the lateral and anterior wall of the head fold and the ectamnion. (**d**) Merged areas according to different contributions.

Figure S3. Regional contribution rate of the amniogenic somatopleure to intraembryonic tissues. A 6×4 square grid with a spacing of 0.25 mm (equivalent to one-sixth of the distance between the anterior tip of the head fold and the anterior edge of somite 1) is superimposed on each side of the template, flanking the embryonic body at the level of mid-hindbrain boundary. Rows are labeled alphabetically (A to F) and columns numerically (–1 to –4 and +1 to +4 for left and right, respectively). The line between rows C and D is positioned at the mid-hindbrain boundary. Each injection site is assigned to a grid cell based on its centroid. Contribution rate (relative percentage of the number of sites contributed to intraembryonic tissues versus that of whole injected sites)

is calculated for each grid cell and displayed as a surface plot using MatlabR2016b (MathWorks) and Illustrator (Adobe). Contribution rates are given in the color grids in the lower panel.

Figure S4. Regional differences in amniogenic somatopleural contribution to intra- and extra-embryonic tissues. Plots of dye-injected sites in the amniogenic somatopleure at 9ss to 15ss (HH10– to HH12–) are categorized according to their contributions (**a**, **d**, **g**, **j**, **m**) and demonstrated with representative images. DiI-injected sites (red arrowheads in **b**, **e**, **h**, **k**, **n**) and later distributions (**c**, **f**, **i**, **l**, **o** in bright field; **c'**, **f'**, **i'**, **l'**, **o'** in dark field) are shown for each category. In images of later distributions, yellow and red arrowheads indicate extents of labeled cells and most intensive fluorescent signals, respectively. am, amnion; ch, chorion; la, left atrium; lb, limb bud; lv, left ventricle; pa1, first pharyngeal arch; pa2, second pharyngeal arch; rv, right ventricle; th, thoracic wall.

Figure S5. Fate analysis of the amniogenic somatopleure by fluorescent-dye injection. (**a**-**f**) Dye-injected sites in the amniogenic somatopleure at 6ss to 8ss (HH9- to HH9+) and distributions at stages equivalent to HH18 to 24 are plotted onto schematic illustrations with different colors as indicated in Fig. S2. A, anterior; P, posterior; L, left; R, right.

Figure S6. Fate mapping via electroporation of the amniogenic somatopleural mesoderm. (**a**) Schematic depiction of the design of electroporation experiments. Plasmid mixture is injected into the space between the left side of head and the somatopleure. The direction of the current flow is right to left as shown in this figure (black arrow). (**b**) Distribution of EGEP fluorescence in the amniogenic somatopleure 7 hours after electroporation (red arrowheads). (**c**) Forty-eight hours after electroporation, EGFP-labeled cells distribute to the amnion (yellow arrowheads) and intraembryonic tissues. (**d**-**i**) Immunostaining for EGFP and the cardiomyocyte marker MHC on transverse sections of the embryo shown in B. Higher magnification images of the boxed regions in **d**-**e** and **g**-**h** were exhibited in **e**-**f** and **h**-**i**, respectively. EGFP-labeled cells distribute to the amnion (yellow arrowheads in **f**), thoracic wall and cardiac outflow tract (yellow arrowheads in **i**). am, amnion; aaf, anterior amniotic fold; fg, foregut; la, left atrium; lv, left ventricle; nt, neural tube; oft, outflow tract; pa1, first pharyngeal arch; pa2, second pharyngeal arch; th, thoracic wall; opv, optic vesicle. Scale bars, 300μm for **d**, **e**, **g** and **h**, 20μm for **f** and **i**.

Figure S7. Destinations of amniogenic somatopleural cells in late stage embryos. (**a**) Dorsal view of a DiI-labeled site (red arrowhead) in the amniogenic somatopleure at the right side of the head fold in a quail embryo at 9ss. (**b**-**d**) Frontal view of the embryo grown to day 8 in bright (**b**) and dark (**c**, **d**) fields. The boxed area in **c** is magnified in **d**. Labeled cells are detected in the midline of the thoracic wall (yellow arrowheads). (**e**-**g**) Frontal view of the same embryo as in **b**-**d** with the thoracic wall removed. Labeled cells distribute along the aorta and to the heart. The white dashed line indicates the level of transvers sections in **h** to **m**. (**h**-**j**) Immunostaining of transverse sections indicated in **e** for the cardiomyocyte marker MHC, merged with DiI fluorescence. DiI-labeled cells are detected in the MHC-positive myocardial layer (yellow arrowheads). Boxed areas in **h** and **i** is magnified in **i** and **j**, respectively. TO-PRO-3 is used to counterstain nuclei. ao, aorta; lv, left ventricle; mb, mandible; pa, pulmonary artery; ra, right atrium; rv, right ventricle; wb, wing bud. Scale bars, 300μm for **h**, 100μm for **i**, 20μm for **j**.

Figure S8. Localization of BMP and FGF gene expression in the pharyngeal arches and outflow tract by in situ hybridization. (**a**-**c**) Hematoxylin-eosin staining of a transverse section of a HH15 chick embryo at the level of the second pharyngeal arch. The boxed region in (**a**) is magnified in (**b**). Anatomical structures are indicated in different colors with possible direction of somatopleural cell migration in (**c**). (**d**-**i**) In situ hybridization on chick embryo sections. Serial sections are derived from chick embryos at HH15 (**d**-**f**) and HH16+ (**g**-**i**). *Bmp2* (**d**, **g**) and *Bmp4* (**e**, **h**) are expressed in the second pharyngeal arch, the outflow tract and the junction between the pericardium and the pharyngeal region. (**f**, **i**) *Fgf8* expresses in ectoderm and endoderm in the second pharyngeal arch. amnion; fg, foregut; oft, outflow tract; pa2; second pharyngeal arch; th, thoracic wall.

Figure S9. Effects of FGF and BMP signaling on endothelial cell differentiation of the amniogenic somatopleure. (**a**-**i**) Immunostaining for the epithelial cell marker cytokeratin and quail-specific vascular endothelial cell marker QH1 on explant culture of the amniogenic somatopleure derived from quail embryos at 7ss to 11ss. Stimulation with FGF2, but not BMP4, induces differentiation of quail somatopleural cells into QH1-positive endothelial cells. TO-PRO-3 is used to counterstain nuclei. Scale bars, 100μm.

Figure S10. FGF signaling induces endothelial cell differentiation of the somatopleure. (**a**-**f**) Immunostaining for the quail-specific vascular endothelial cell marker QH1 on explant culture of the amniogenic somatopleure derived from quail embryos at 7ss to 11ss. The induction of endothelial cell differentiation by FGF2 (**c**, **d**) is inhibited by FGF receptor antagonist SU5402 (**e**, **f**). TO-PRO-3 is used to counterstain nuclei. Scale bars, 100μm.

Figure S11. BMP signaling induces cardiomyocyte differentiation of the amniogenic somatopleure. (**a**-**i**) Immunostaining of the cardiac progenitor cell marker Nkx2.5 and cardiomyocyte marker myosin heavy chain (MHC) on explant culture of the somatopleure derived from quail embryos in ss7 to ss11. Stimulation with BMP4 induces differentiation of quail somatopleural cells into cardiomyocytes (**d**-**f**), which is inhibited by Noggin, a BMP antagonist (**g**-**i**). TO-PRO-3 is used to counterstain nuclei. Scale bars, 20μm.

Figure S12. BMP signaling from the pharyngeal arches induces differentiation of somatopleure into cardiomyocytes. (**a**-**i**) Amniogenic somatopleural explants from quail embryos in ss9 to ss11 were co-cultured with explants of the chicken first and second pharyngeal arches (pa1/pa2) at HH15 and were immunostained for the quail cell-specific marker QCPN and the cardiac progenitor cell marker Nkx2.5. Co-culture with pharyngeal arch explants induces cardiomyocyte differentiation in the somatopleural explants (**d**-**f**). Noggin suppressed the induction of cardiomyocyte differentiation (**g**-**i**), indicating the involvement of BMP signaling. TO-PRO-3 is used to counterstain nuclei. Scale bars, 20μm.

Fig. S1

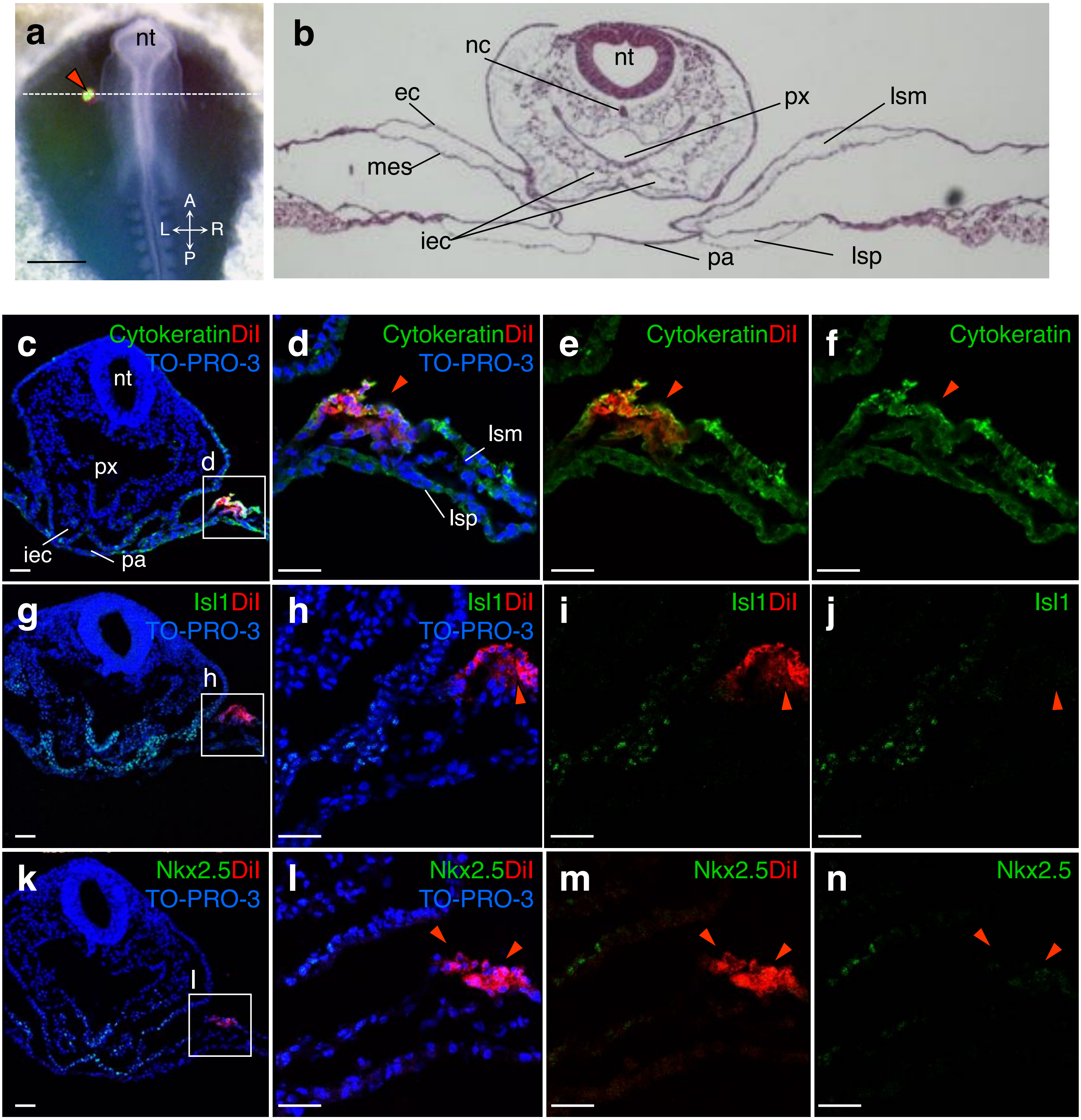
a
nt
A
L
R
P
b
nc
nt
ec
px
lsm
mes
iec
pa
lsp
c
CytokeratinDiI
TO-PRO-3
nt
px
d
iec
pa
d
CytokeratinDiI
TO-PRO-3
lsm
lsp
e
CytokeratinDiI
f
Cytokeratin
g
Isl1DiI
TO-PRO-3
h
h
Isl1DiI
TO-PRO-3
i
Isl1DiI
j
Isl1
k
Nkx2.5DiI
TO-PRO-3
l
l
Nkx2.5DiI
TO-PRO-3
m
Nkx2.5DiI
n
Nkx2.5

Fig. S2

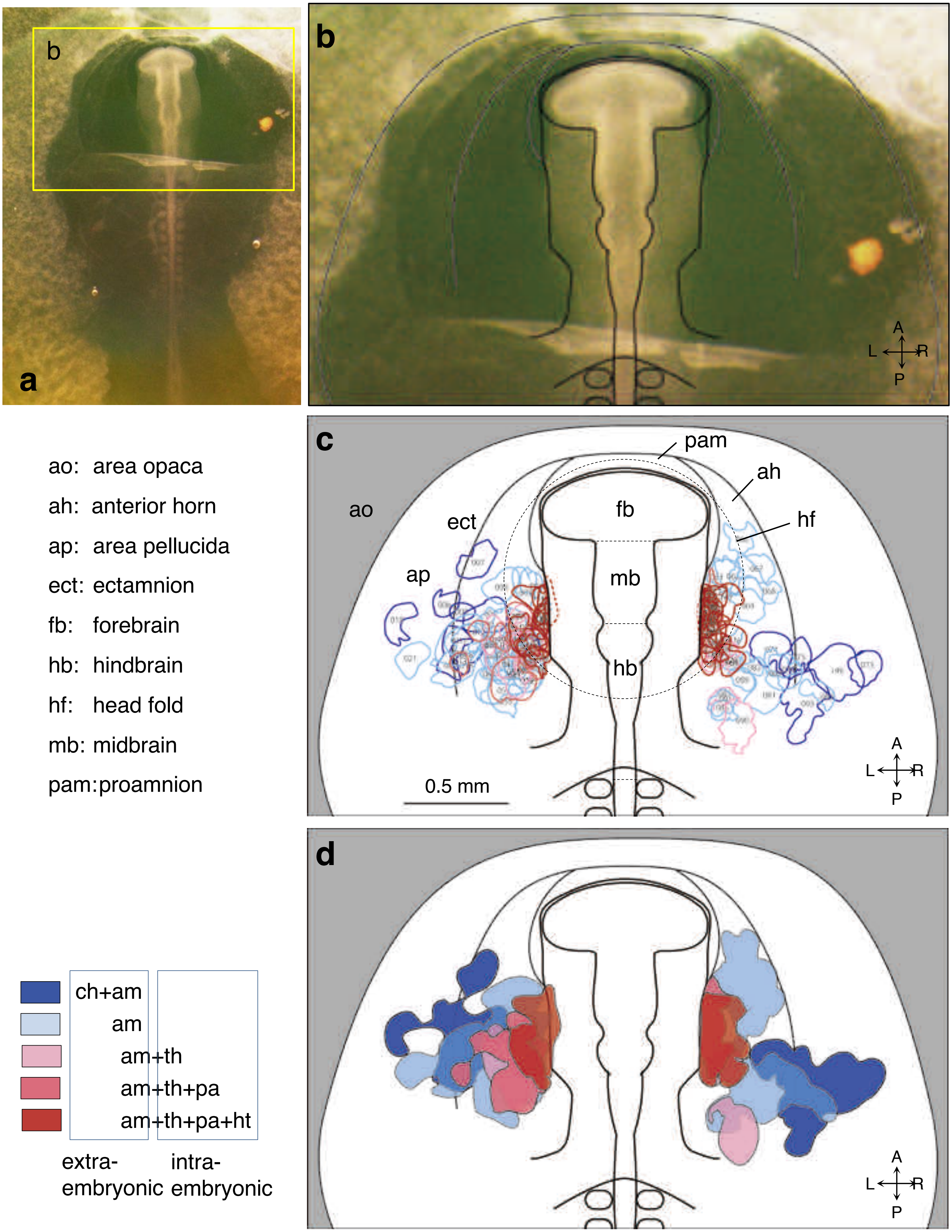
a
b
c
d
A
L
R
P
ao
ect
ap
pam
ah
hf
fb
mb
hb
0.5 mm
ao: area opaca
ah: anterior horn
ap: area pellucida
ect: ectamnion
fb: forebrain
hb: hindbrain
hf: head fold
mb: midbrain
pam:proamnion
ch+am
am
am+th
am+th+pa
am+th+pa+ht
extra-
embryonic
intra-
embryonic

Fig. S3

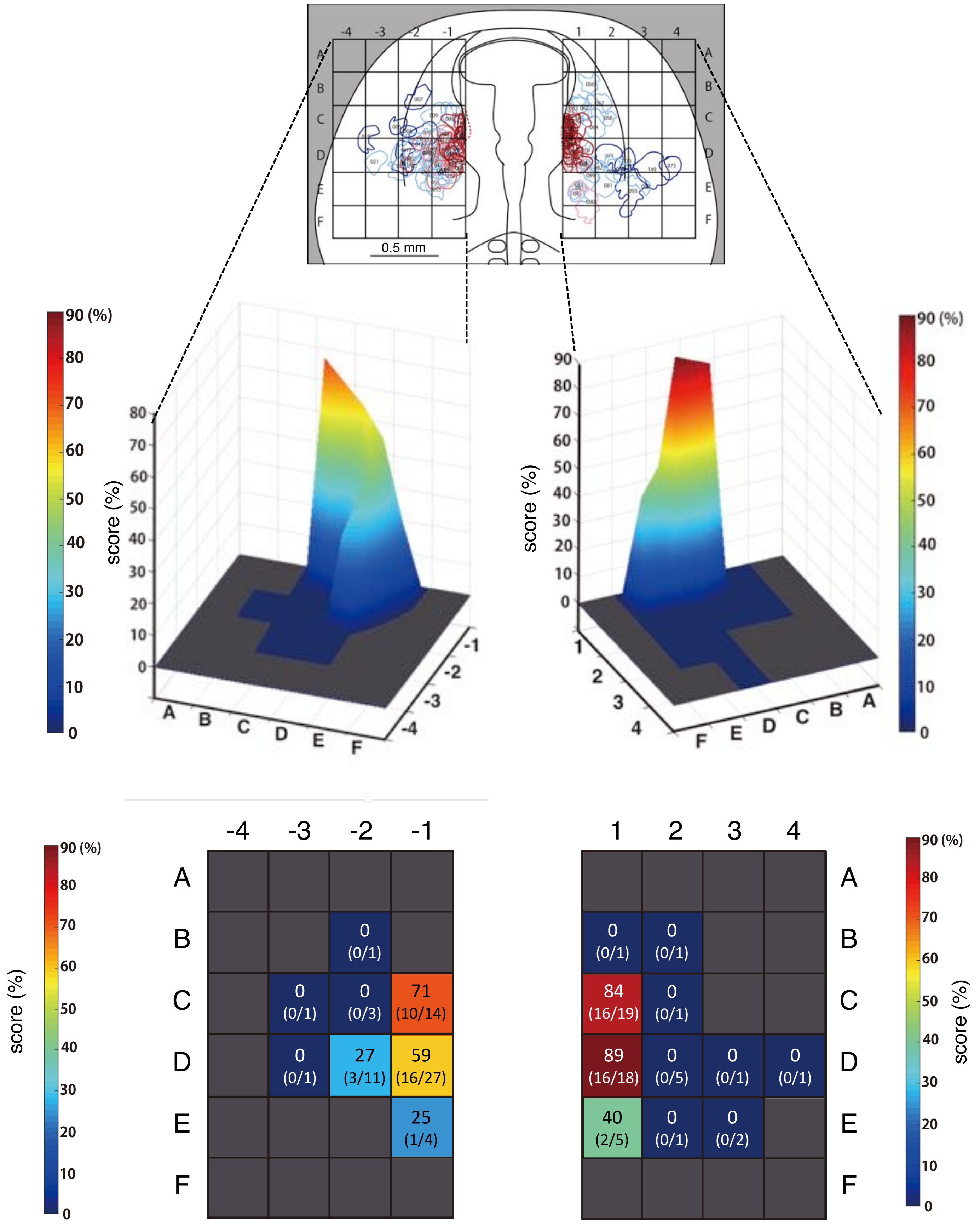

-4 -3 -2 -1 1 2 3 4
A B C D E F
0.5 mm
90 (%)
80
70
60
50
40
30
20
10
0
score (%)
A B C D E F
-1 -2 -3 -4
1 2 3 4
F E D C B A
-4 -3 -2 -1
A B C D E F
B-2: 0 (0/1)
C-3: 0 (0/1)
C-2: 0 (0/3)
C-1: 71 (10/14)
D-3: 0 (0/1)
D-2: 27 (3/11)
D-1: 59 (16/27)
E-1: 25 (1/4)
1 2 3 4
B1: 0 (0/1)
B2: 0 (0/1)
C1: 84 (16/19)
C2: 0 (0/1)
D1: 89 (16/18)
D2: 0 (0/5)
D3: 0 (0/1)
D4: 0 (0/1)
E1: 40 (2/5)
E2: 0 (0/1)
E3: 0 (0/2)

Fig. S4

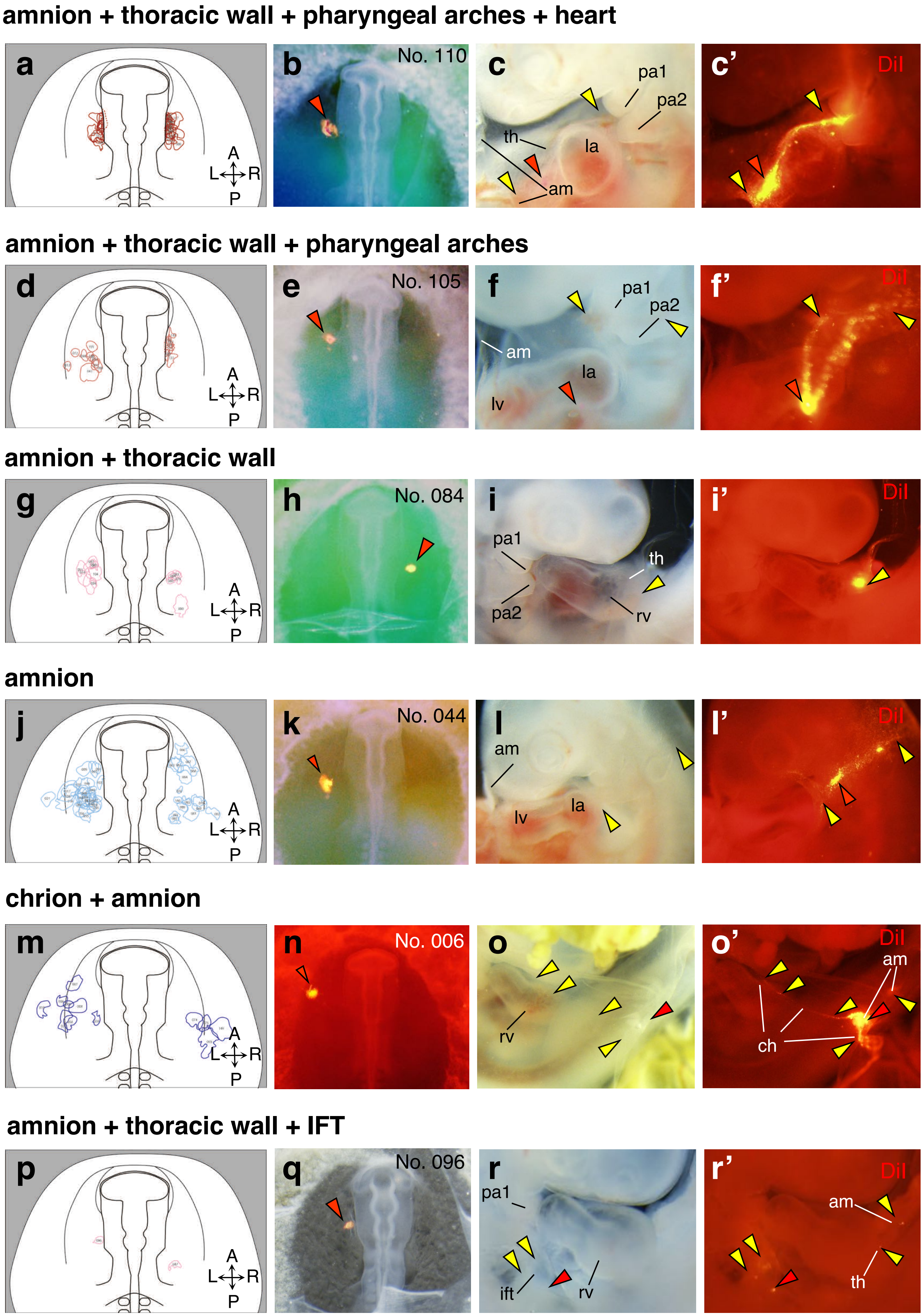
amnion + thoracic wall + pharyngeal arches + heart
a
A
L
R
P
b
No. 110
c
pa1
pa2
th
la
am
c'
DiI
amnion + thoracic wall + pharyngeal arches
d
A
L
R
P
e
No. 105
f
pa1
pa2
am
la
lv
f'
DiI
amnion + thoracic wall
g
A
L
R
P
h
No. 084
i
pa1
th
pa2
rv
i'
DiI
amnion
j
A
L
R
P
k
No. 044
l
am
la
lv
l'
DiI
chrion + amnion
m
A
L
R
P
n
No. 006
o
rv
o'
DiI
am
ch
amnion + thoracic wall + IFT
p
A
L
R
P
q
No. 096
r
pa1
ift
rv
r'
DiI
am
th

Fig. S5

6ss to 8ss

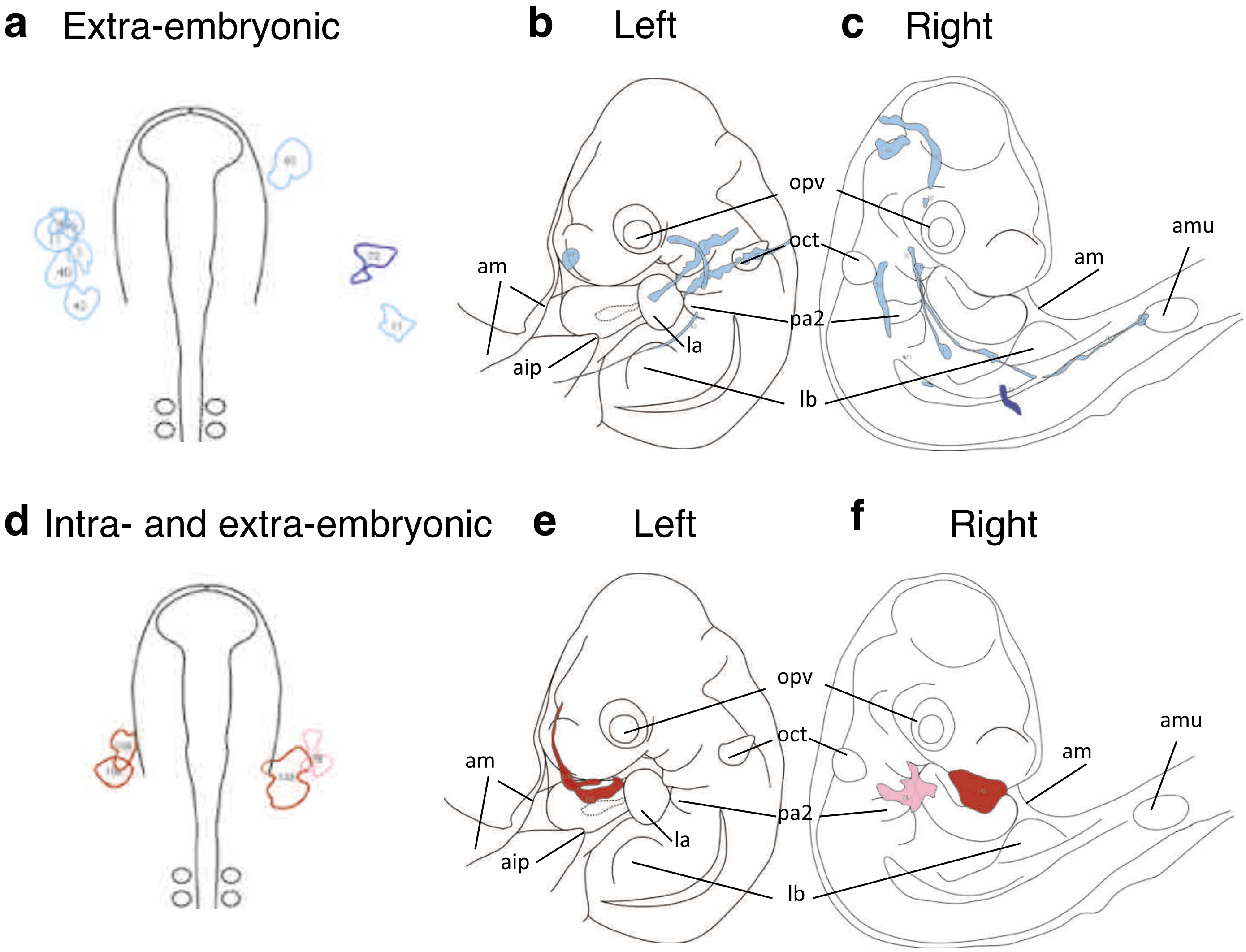

Fig. S6

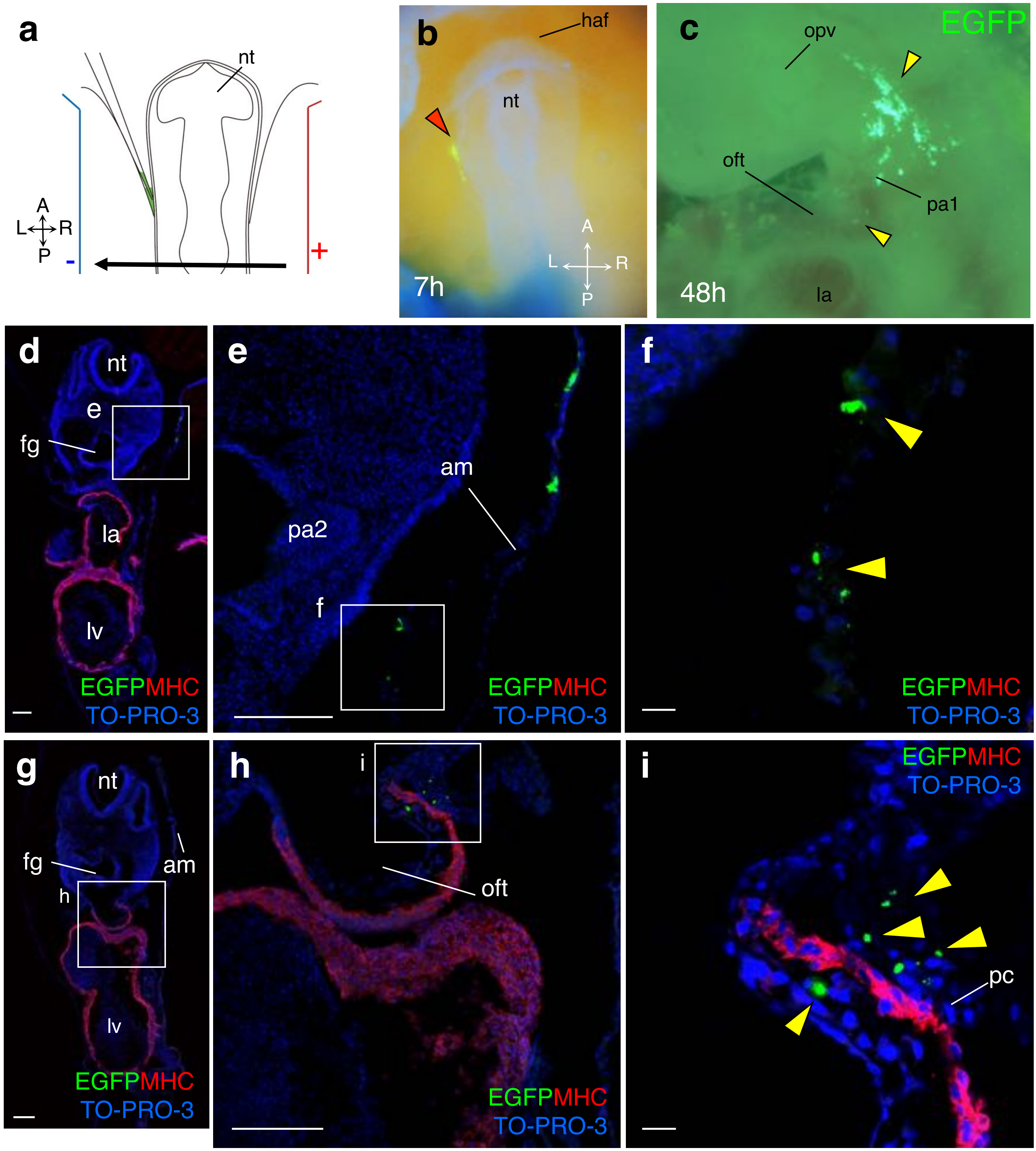
a
nt
A
L
R
P
-
+
b
haf
nt
A
L
R
P
7h
c
EGFP
opv
oft
pa1
48h
la
d
nt
e
fg
la
lv
EGFPMHC
TO-PRO-3
e
am
pa2
f
EGFPMHC
TO-PRO-3
f
EGFPMHC
TO-PRO-3
g
nt
fg
am
h
lv
EGFPMHC
TO-PRO-3
h
i
oft
EGFPMHC
TO-PRO-3
i
EGFPMHC
TO-PRO-3
pc

Fig. S7

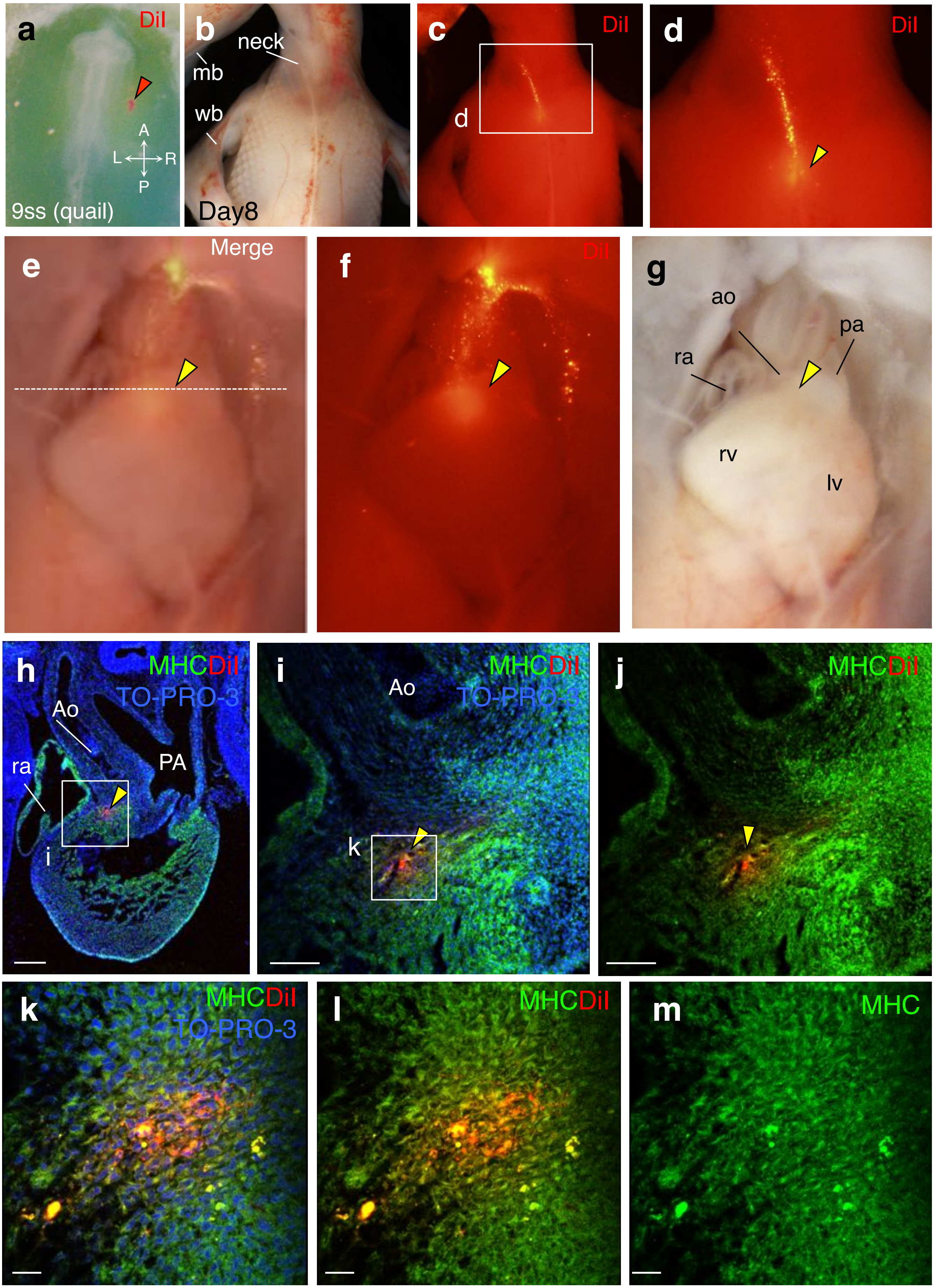

a
DiI
A
L
R
P
9ss (quail)
b
neck
mb
wb
Day8
c
DiI
d
d
DiI
e
Merge
f
DiI
g
ao
pa
ra
rv
lv
h
MHCDiI
TO-PRO-3
Ao
ra
PA
i
i
MHCDiI
TO-PRO-3
Ao
k
j
MHCDiI
k
MHCDiI
TO-PRO-3
l
MHCDiI
m
MHC

Fig. S8

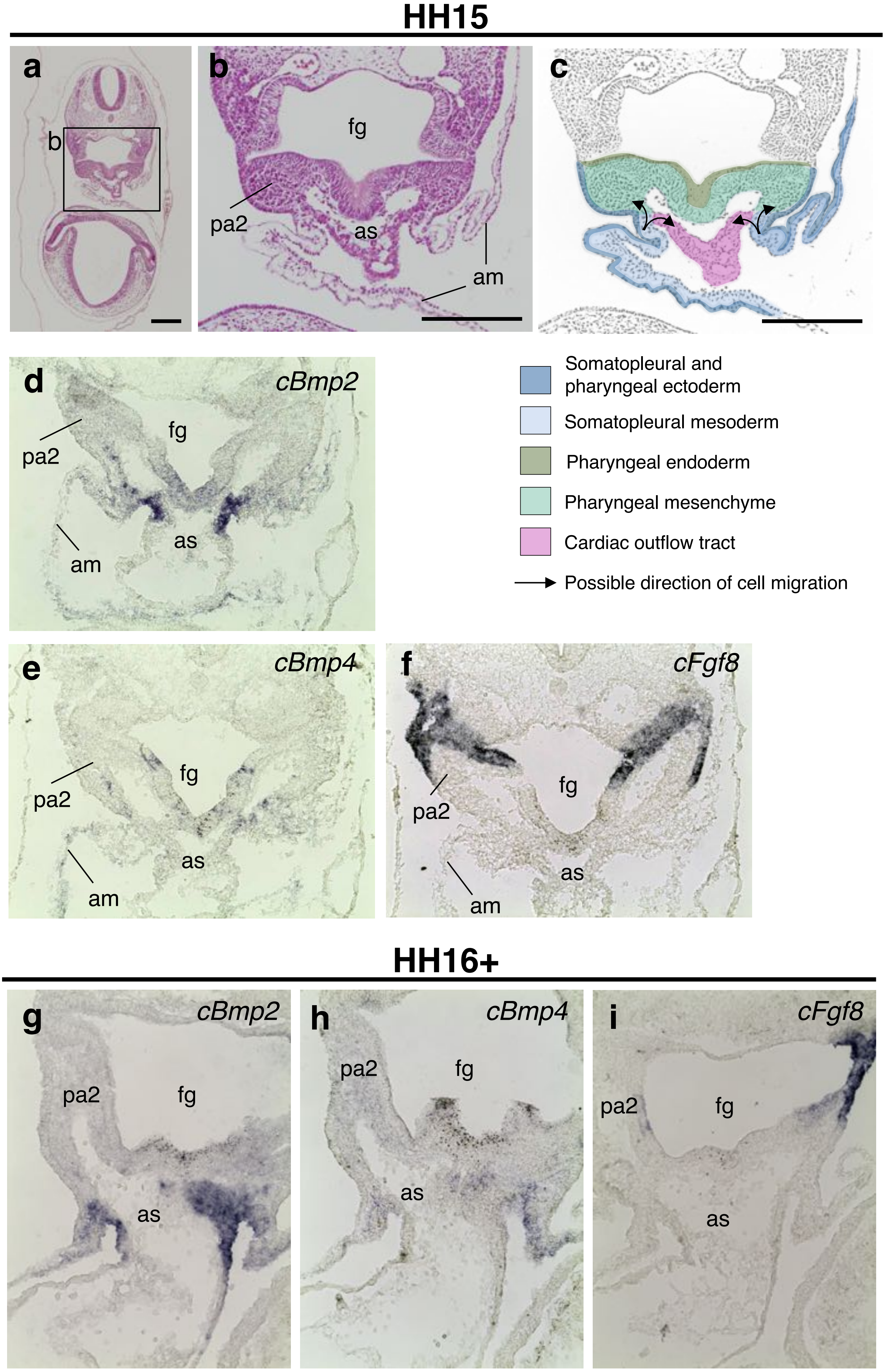

HH15
a
b
c
fg
pa2
as
am
d
cBmp2
Somatopleural and pharyngeal ectoderm
Somatopleural mesoderm
Pharyngeal endoderm
Pharyngeal mesenchyme
Cardiac outflow tract
Possible direction of cell migration
e
cBmp4
f
cFgf8
HH16+
g
h
i

Fig. S9

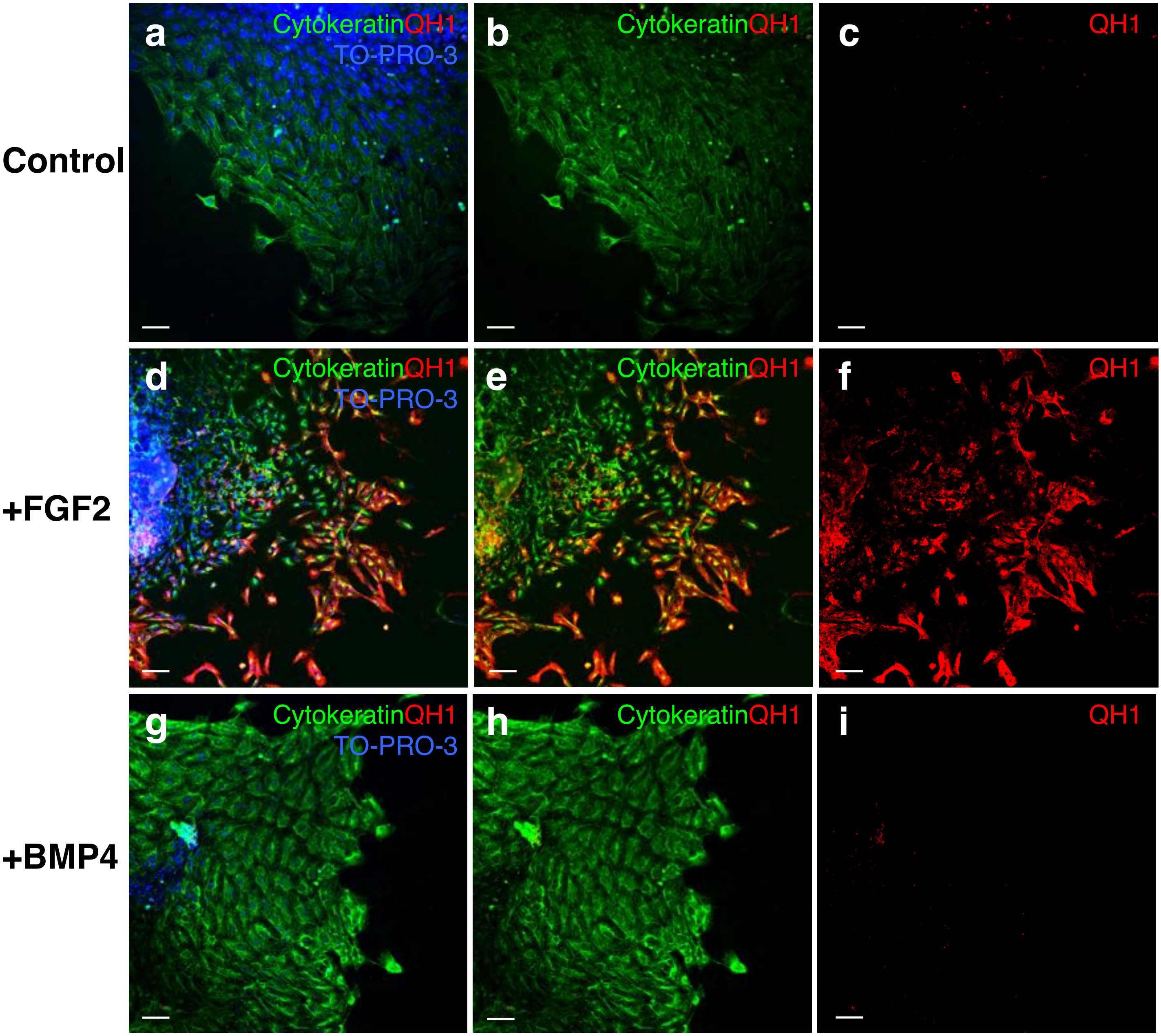
Control
+FGF2
+BMP4
a
CytokeratinQH1
TO-PRO-3
b
CytokeratinQH1
c
QH1
d
CytokeratinQH1
TO-PRO-3
e
CytokeratinQH1
f
QH1
g
CytokeratinQH1
TO-PRO-3
h
CytokeratinQH1
i
QH1

Fig. S10

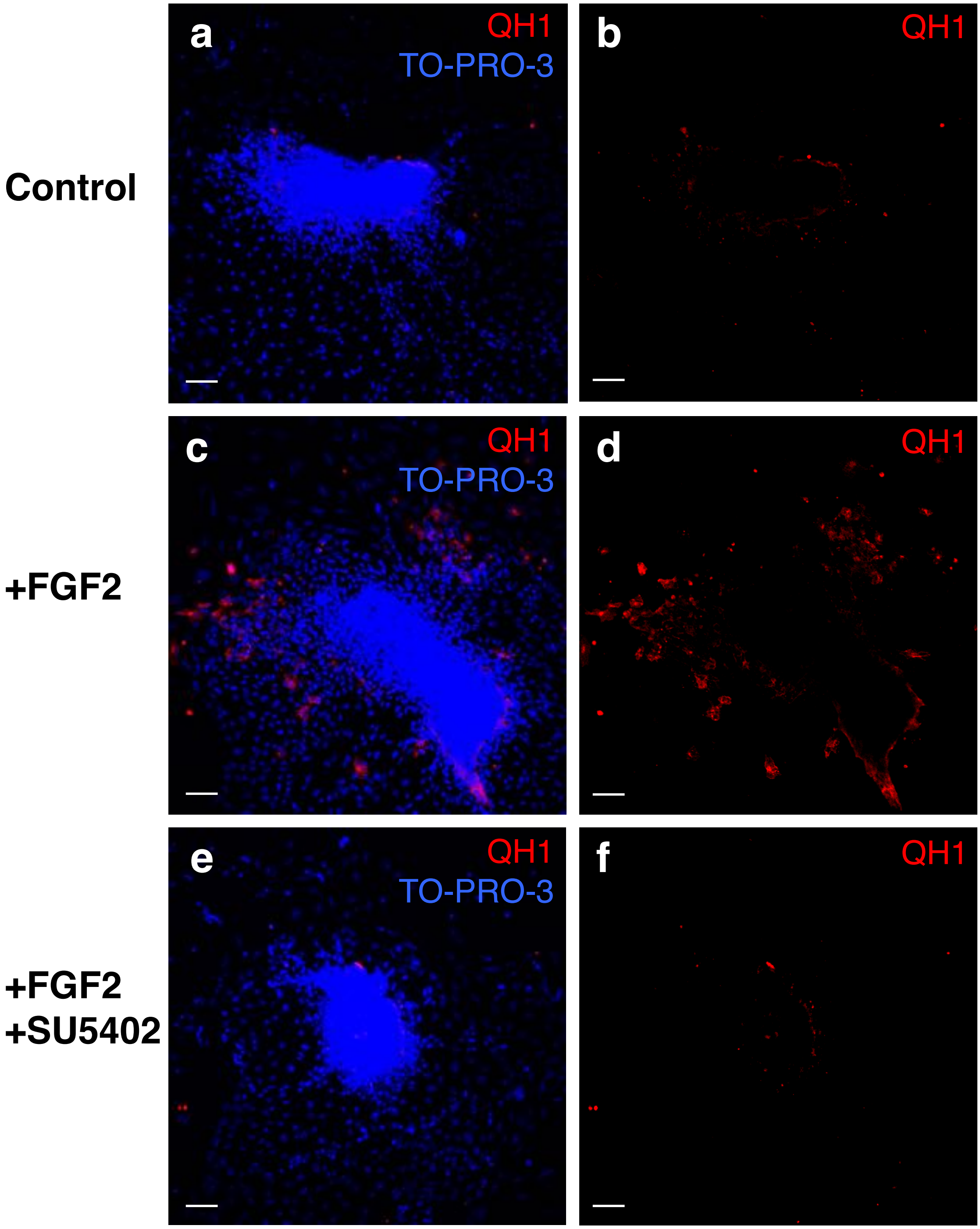
Control
+FGF2
+FGF2
+SU5402
a
QH1
TO-PRO-3
b
QH1
c
QH1
TO-PRO-3
d
QH1
e
QH1
TO-PRO-3
f
QH1

Fig. S11

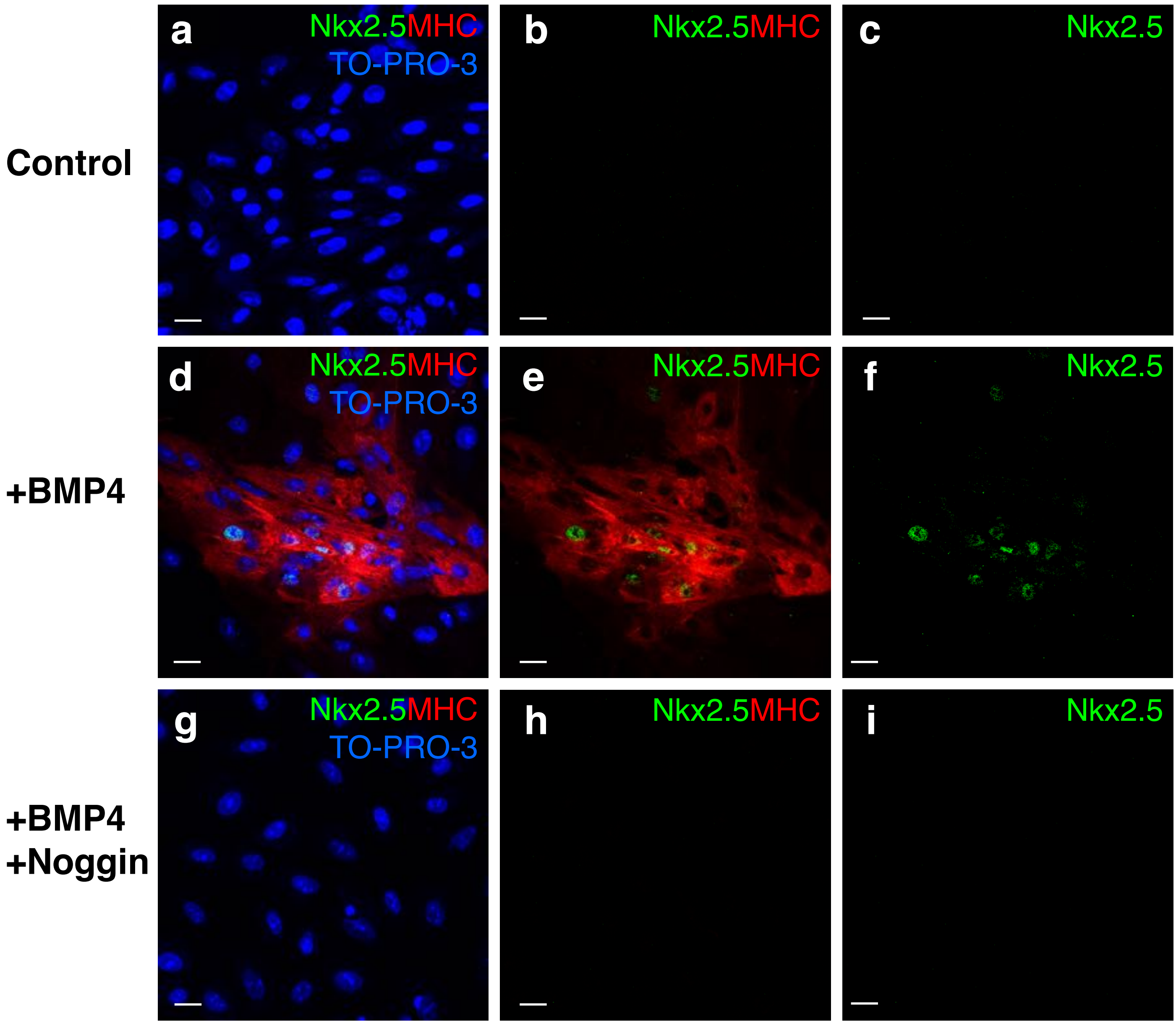

a
Nkx2.5MHC
TO-PRO-3
b
Nkx2.5MHC
c
Nkx2.5
Control
d
Nkx2.5MHC
TO-PRO-3
e
Nkx2.5MHC
f
Nkx2.5
+BMP4
g
Nkx2.5MHC
TO-PRO-3
h
Nkx2.5MHC
i
Nkx2.5
+BMP4
+Noggin

Fig. S12

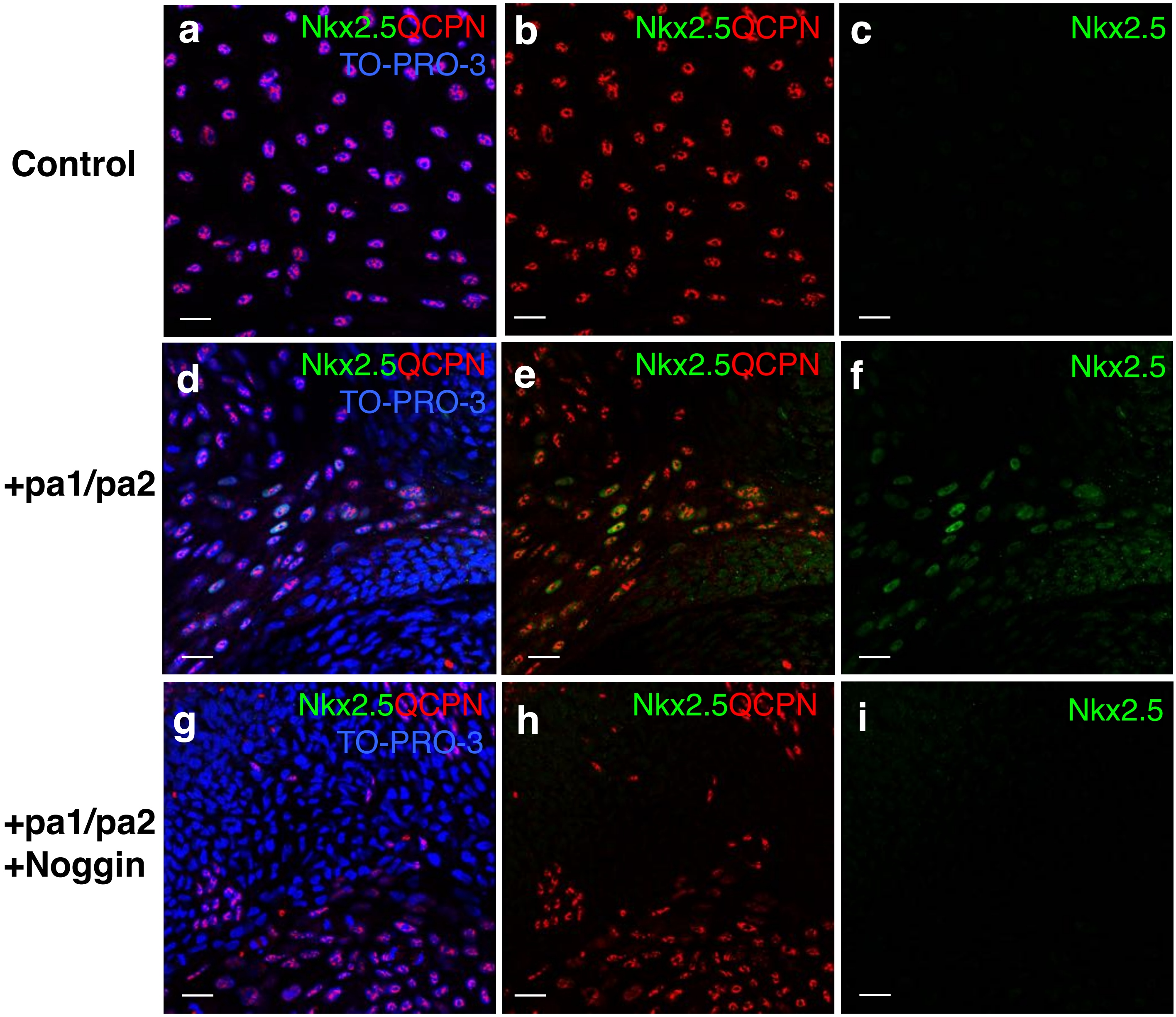
Control
+pa1/pa2
+pa1/pa2
+Noggin
a
Nkx2.5QCPN
TO-PRO-3
b
Nkx2.5QCPN
c
Nkx2.5
d
Nkx2.5QCPN
TO-PRO-3
e
Nkx2.5QCPN
f
Nkx2.5
g
Nkx2.5QCPN
TO-PRO-3
h
Nkx2.5QCPN
i
Nkx2.5

Table S1. Summary of dye-labeling experiments with classification according to final contributions.

mapping n	somite	contribution	sample stage
4	8	am/ch	HH18
5	11	am/ch	HH18
6	11	am/ch	HH18
7	11	am/ch	HH18
8	9	am/ch	HH18
19	13	am/ch	HH18
20	12	am/ch	HH18
39	14	am/ch	HH20
58	11	am/ch	HH18
73	11	ch	Day4(HH24)
74	10	am/ch	Day4(HH24)
75	10	am/ch	Day4(HH24)
93	11	am/ch	HH18
149	10	am/ch	HH19
1	10	am	HH18
2	8	am	HH18
3	10	am	Day4(HH24)
9	10	am	HH18
10	8	am	HH18
11	8	am	HH18
16	7	am	HH18
17	11	am	HH18
18	10	am	HH18
21	10	am	HH18
29	12	am	HH18
30	11	am	HH18
37	9	am	HH23
38	10	am	HH24
40	6	am	HH18
42	8	am	HH18
43	9	am	HH18
44	10	am	HH18
45	10	am	HH18
46	10	am	Day4(HH24)
47	8	am	Day4(HH24)
48	11	am	Day4(HH24)
49	11	am	Day4(HH24)
50	9	am	HH18
51	12	am	HH19
53	8	am	HH18
54	14	am	HH18
55	14	am	HH19
56	9	am	HH18
60	10	am	HH20
62	10	am	HH18
63	9	am	HH18
64	10	am	HH21
65	8	am	HH21
66	11	am	Day4(HH24)
67	9	am	Day4(HH24)
68	10	am	HH18
69	9	am	HH18
70	12	am	HH18
71	8	am	HH20
72	8	am	HH20
76	10	am	HH18
81	11	am	HH18
82	11	am	HH19
85	9	am	HH18
88	8	am	HH18
89	11	am	HH24
91	12	am	HH21
92	10	am	HH18
102	10	am	Day4(HH22)
103	10	am	Day4(HH24)
107	9	am	HH20
146	12	am	HH20

mapping n	somite	contribution	sample stage
14	10	am/th	HH18
31	11	am/th	HH19
36	8	am/th	HH18
59	8	am/th	Day4(HH25)
78	8	am/th	HH18
79	11	am/th	HH18
80	10	am/th	HH24
83	10	am/th	HH19
84	9	am/th	HH18
87	10	am/th/IFT	HH18
90	12	am/th	HH21
94	10	am/th	HH18
95	12	am/th	HH19
96	15	am/IFT	HH19
97	13	am/th	HH19
104	12	am/th	HH18
12	11	am/PA1	HH18
13	10	am/th/PA2	HH18
15	8	am/th/PA1	HH19
32	13	am/th/PA2	HH19
33	9	am/th/PA2	HH18
35	13	am/th/PA1/PA2	HH18
41	9	am/th/PA2	HH18
61	10	am/th/PA1	HH20
98	11	am/th/PA1/PA2	HH19
99	12	am/th/PA1	HH19
100	13	am/th/PA1/PA2	HH18
105	9	am/th/PA1/PA2	HH18
109	12	am/th/PA1/PA2	HH19
121	8	am/th/PA1	HH18
124	10	am/th/PA1	HH19
131	11	am/th/PA1	HH19
132	11	am/th/PA1	HH19
133	10	am/th/PA1	HH19
137	11	am/th/PA1	HH19
141	11	am/th/PA1	HH19
143	10	am/th/PA1	HH19
86	13	am/th/PA2/OFT	HH19
101	11	am/th/PA1/PA2/OFT	HH18
106	7	am/th/PA1/PA2/OFT	HH20
108	7	am/th/PA2/OFT	HH20
110	13	am/th/PA1/PA2/OFT	HH18
111	11	am/th/PA1/OFT	HH18
112	8	am/th/PA1/PA2/OFT	HH19
113	11	am/th/PA1/OFT	HH19
114	10	am/th/PA1/OFT	HH19
115	9	am/th/PA1/PA2/OFT	HH19
116	9	am/th/PA1/PA2/OFT	HH19
117	10	am/th/PA1/OFT	HH19
118	9	am/th/PA1/OFT	HH19
119	9	am/th/PA1/OFT	HH19
120	9	am/th/PA1/OFT	HH19
122	10	am/th/PA1/OFT	HH19
123	10	am/th/PA1/OFT	HH19
125	10	am/th/PA1/PA2/OFT	HH19
126	10	am/th/PA1/OFT	HH19
127	10	am/th/PA1/OFT	HH19
128	10	am/th/PA1/PA2/OFT	HH19
130	11	am/th/PA1/OFT	HH19
134	10	am/th/PA1/OFT	HH19
135	11	am/th/PA1/PA2/OFT	HH19
136	11	am/th/PA1/OFT	HH19
138	10	am/th/PA1/PA2/OFT	HH19
139	11	am/th/PA1/PA2/OFT	HH19
140	11	am/th/PA1/PA2/OFT	HH19
142	11	am/th/PA1/OFT	HH19
144	10	am/th/PA1/PA2/OFT	HH19
145	9	am/PA1/PA2/OFT	HH24
147	11	am/th/PA1/OFT	HH20
148	8	am/th/PA1/PA2/OFT	HH18
150	12	am/th/PA1/PA2/OFT	Day4
151	12	am/th/PA1/PA2/OFT	Day4

am/ch
am
am/th(/IFT)
am/th/PA
am/th/PA/OFT

am: amnion
ch: chorion
IFT: cardiac inflow tract
OFT: cardiac outflow tract
PA: pharyngeal arch
  PA1: 1st pharyngeal arch
  PA2: 2nd pharyngeal arch
th: thoracic wall
